# Population-based genomic study of *Plasmodium vivax* malaria in seven Brazilian states and across South America

**DOI:** 10.1016/j.lana.2022.100420

**Published:** 2023-01-02

**Authors:** Amy Ibrahim, Emilia Manko, Jamille G. Dombrowski, Mónica Campos, Ernest Diez Benavente, Debbie Nolder, Colin J. Sutherland, Francois Nosten, Diana Fernandez, Gabriel Vélez-Tobón, Alberto Tobón Castaño, Anna Caroline C. Aguiar, Dhelio Batista Pereira, Simone da Silva Santos, Martha Suarez-Mutis, Silvia Maria Di Santi, Andrea Regina de Souza Baptista, Ricardo Luiz Dantas Machado, Claudio R.F. Marinho, Taane G. Clark, Susana Campino

**Affiliations:** a Faculty of Infectious & Tropical Diseases, London School of Hygiene & Tropical Medicine, London, UK; b Department of Parasitology, Institute of Biomedical Sciences, University of São Paulo, São Paulo, Brazil; c Public Health England Malaria Reference Laboratory, London School of Hygiene & Tropical Medicine, London, UK; d Shoklo Malaria Research Unit, Mahidol-Oxford Tropical Medicine Research Unit, Faculty of Tropical Medicine, Mahidol University, Mae Sot, Tak, Thailand; e Centre for Tropical Medicine and Global Health, Nuffield Department of Clinical Medicine Research Building, University of Oxford Old Road Campus, Oxford, UK; f Grupo Malaria, Facultad de Medicina, Universidad de Antioquia, Antioquia, Colombia; g Department of Bioscience, Federal University of São Paulo, São Paulo, Brazil; h Research Center for Tropical Medicine of Rondonia, Porto Velho, Brazil; i Laboratório de Doenças Parasitárias, Institute Oswaldo Cruz - Fiocruz- Rio de Janeiro, Brazil; j School of Medicine, University of São Paulo, Brazil; k Centro de Investigação de Microrganismos – CIM, Departamento de Microbiologia e Parasitologia, Universidade Federal Fluminense, Brazil; l Faculty of Epidemiology & Population Health, London School of Hygiene & Tropical Medicine, London, UK

**Keywords:** Malaria, Plasmodium, Plasmodium vivax, Non-falciparum malaria, Brazil, South America, Whole genome sequencing, Population genetics, Vector-borne diseases, Drug resistance, Genomics

## Abstract

**Background:**

Brazil is a unique and understudied setting for malaria, with complex foci of transmission associated with human and environmental conditions. An understanding of the population genomic diversity of *P. vivax* parasites across Brazil can support malaria control strategies.

**Methods:**

Through whole genome sequencing of *P. vivax* isolates across 7 Brazilian states, we use population genomic approaches to compare genetic diversity within country (n = 123), continent (6 countries, n = 315) and globally (26 countries, n = 885).

**Findings:**

We confirm that South American isolates are distinct, have more ancestral populations than the other global regions, with differentiating mutations in genes under selective pressure linked to antimalarial drugs (*pvmdr1*, *pvdhfr-ts*) and mosquito vectors (*pvcrmp3, pvP45/48, pvP47*). We demonstrate Brazil as a distinct parasite population, with signals of selection including ABC transporter (*PvABCI3*) and PHIST exported proteins.

**Interpretation:**

Brazil has a complex population structure, with evidence of *P. simium* infections and Amazonian parasites separating into multiple clusters. Overall, our work provides the first Brazil-wide analysis of *P. vivax* population structure and identifies important mutations, which can inform future research and control measures.

**Funding:**

AI is funded by an 10.13039/501100018814MRC LiD PhD studentship. TGC is funded by the 10.13039/501100000265Medical Research Council (Grant no. MR/M01360X/1, MR/N010469/1, MR/R025576/1, MR/R020973/1 and MR/X005895/1). SC is funded by 10.13039/501100000265Medical Research Council UK grants (MR/M01360X/1, MR/R025576/1, MR/R020973/1 and MR/X005895/1) and Bloomsbury SET (ref. CCF17-7779). FN is funded by The Shloklo Malaria Research Unit - part of the Mahidol Oxford Research Unit, supported by the 10.13039/100010269Wellcome Trust (Grant no. 220211). ARSB is funded by 10.13039/501100001807São Paulo Research Foundation - FAPESP (Grant no. 2002/09546–1). RLDM is funded by 10.13039/501100003593Brazilian National Council for Scientific and Technological Development - CNPq (Grant no. 302353/2003–8 and 471605/2011–5); CRFM is funded by 10.13039/501100001807FAPESP (Grant no. 2020/06747–4) and 10.13039/501100003593CNPq (Grant no. 302917/2019–5 and 408636/2018–1); JGD is funded by 10.13039/501100001807FAPESP fellowships (2016/13465–0 and 2019/12068–5) and 10.13039/501100003593CNPq (Grant no. 409216/2018–6).

**Research in context**

**Evidence before this study**

Human malaria caused by infections with *P. vivax* parasites are a significant threat to global health. Whilst less pathogenic than *P. falciparum*, *P. vivax* infections are more widely spread, with one third of the world's population at risk of infection, resulting in a huge impact on morbidity. Prior to this study, whole genome sequencing (WGS) of Brazilian *P. vivax* isolates has focussed on the two states of Amazonas and Acre, both in the Amazonian region. These studies demonstrated genetically diverse populations of parasites within the Americas, signs of inbreeding in high-transmission sites in Brazil such as Mancio Lima, and evidence of mutations within genes associated with drug susceptibility. There are however other transmission foci within Brazil and the genomic background of these foci has yet to be investigated.

**Added value of this study**

This study analyses high-quality genome data from 885 clinical isolates of *P. vivax* sourced globally, including 315 from South America, and 123 from Brazil covering seven states. Using the largest genomic dataset of Brazilian *P. vivax* isolates, we demonstrate a complex population structure at country level, with a distinct population of isolates from São Paulo that may be *P. simium.* We find Brazil-specific mutations in genes associated with mosquito life stages and drug susceptibility and suggest potential novel candidates for further investigation.

**Implications of all the available evidence**

Malaria continues to be an important public health issue in Brazil. To tackle this disease effectively, it is crucial to understand the genetic make-up of the underlying parasites. Through WGS studies, it is possible to identify genetic differences between populations that may enable us to target parasites more effectively. By screening drug resistance markers, it is possible to determine the most effective treatment regimen to use. Here, we investigate the largest genomic dataset of clinical *P. vivax* isolates in Brazil, and determine mutations within genes associated with drug susceptibility, alongside Brazilian-specific variants in genes associated with mosquito transmission stages, potentially informing an understanding of transmission in the country and the wider region.

## Introduction

The *Plasmodium vivax* parasite causes the highest malaria burden outside of sub-Saharan Africa,^1^ with more than one third of the global population at risk due to its wide geographical range.^2^ Complications associated with *P. vivax* infections can lead to severe, life-threatening syndromes.^3^ *P. vivax* infections underly the majority (>80%) of the >700k annual malaria cases in the Americas, including in South America, where countries surrounding the Amazon rain forest areas, such as Brazil, Colombia, and Venezuela, have hotspots of endemic disease.^1^ In South America, malaria transmission dynamic studies are complicated by *P. vivax* and *P. falciparum* co-infections, which have differences in their life cycles and transmission patterns.^1^ Further, significant challenges continue to thwart *P. vivax* control, including the ability of parasites to form dormant hypnozoite stages within the liver, leading to relapses of malaria if not treated using a radical cure of primaquine.^4^ Unfortunately, individuals with glucose-6-phosphate dehydrogenase deficiency are at risk of developing severe haemolysis if treated with primaquine or tafenoquine, and therefore along with pregnant women and infants, are ineligible for radical cure.^5^ Additionally, control measures are compromised by the presence of sub-microscopic and asymptomatic *P. vivax* infections, leading to untreated human parasite reservoirs.^6^ Human settlement and mobility, including through peri-urban expansion, gold mining-related activities, and deforestation in the Amazon, all lead to significantly higher risk of malaria infections.^5^^,^^7^ In addition, self-medication with poor adherence, reported in these regions, can contribute to relapses of *P. vivax* infections and may contribute to selection of mutations leading to parasite drug resistance.^8^^,^^9^ There are many gaps in our knowledge of *P. vivax* infections, including malaria in pregnancy which is understudied, and a lack of knowledge of genetic markers for drug resistance, specifically regarding the first line antimalarial, chloroquine, to which resistance has emerged in many countries including South America,^10^ calling for a rapid change in *P. vivax* control strategy.

Brazil has a diverse geographical profile leading to variation in malaria transmission, with foci split into three discrete groups, each with unique settings for transmission. First, the Amazon rainforest in the northeast of Brazil, which accounts for 99% of all malaria cases, and transmission is led by *Anopheles darlingi* and *An. albitarsis* complex mosquitoes. Second, the north-western coastal border of Brazil, where transmission is lower and due to the *An. aquasalis* mosquito species. Third, the Atlantic Rainforest on the south-western coastal border of the country, where transmission is mainly mediated by *An. bellator* and *An. cruzii*.^11^ In this southern region, *P. simium* is transmitted by *An. cruzii,* and is genetically highly related to *P. vivax*. *P. simium* is found mainly in non-human primates^12^ but human cases have been recorded in São Paulo and Rio de Janeiro.^13^ Within Brazil, there is both inter-state transmission and importation of malaria from neighbouring countries. Due to their proximity, countries bordering the Amazonian region (French Guiana, Guyana, Venezuela, and Peru) play an important role in malaria transmission in the Amazon. Importation of malaria, whether between countries, or within country is a threat to elimination, as malaria-free regions neighbouring those with malaria transmission are at a constant risk of importation and resulting outbreaks.^11^

Human genetics may contribute to *P. vivax* transmission dynamics in Brazil, with the presence of the Duffy negative (Fy-) blood group phenotype, which hinders *P. vivax* invasion of human erythrocytes.^14^ The Fy-phenotype appears to be more common on the north-western coast of Brazil, with some areas demonstrating >50% frequency.^15^ In contrast, the phenotype appears less common in studies based in the Amazonian region, where vivax-malaria transmission is highest. For example, the Fy-phenotype frequency is only 2.8% in Presidente Figueiredo, where malaria transmission is high (annual parasite index (API) of 301.65 malaria cases per 1000 individuals).^16^ Whilst Fy-was previously thought to provide complete protection to *P. vivax* infection, there have been reports,^17^, ^18^, ^19^, ^20^ including in Brazil,^21^ that vivax-infections can occur in Fy-individuals. Furthermore, there are concerns that vivax-malaria in Fy-individuals may present as an asymptomatic infection with lower asexual parasitaemias than Duffy positive individuals, which could lead to a large silent parasite reservoir, complicating malaria eradication.^22^^,^^23^

In contrast to the wider transmission of *P. vivax* in Brazil, *P. falciparum* infections are restricted to hotspot areas, mostly found within the Amazonian rainforest states of Amazonas and Acre, the two states which account for >45% of all malaria cases.^24^ Control measures for malaria have been designed against *P. falciparum* infections and are widely known to be less effective at tackling *P. vivax*, due to key differences in parasite biology. For example, *P. vivax* parasites are able to create dormant liver stage parasites, known as hypnozoites, which are not cleared using routine antimalarials, and require additional treatment, known as radical cure.^25^ Additionally, *P. vivax* parasites are viable within a wider temperature range than *P. falciparum,* allowing for their spread into a greater geographical area,^26^ with transmission further aided by the permissibility of *P. vivax* to multiple mosquito vector species.^27^

In Brazil, chloroquine is still used to treat *P. vivax* infections, even though resistance has already been documented in both *P. falciparum* and *P. vivax* parasites.^10^ Surveillance of drug resistance in *P. vivax* parasites is a challenge as the underlying genetic markers for resistance are unknown. Whole genome sequencing (WGS) of *P. vivax* could provide insights into genetic mutations underlying both drug resistance and population structure. Studies have shown that *P. vivax* parasites within South America display high levels of genetic diversity, comparable to high transmission regions such as Southeast Asia.^28^ This difference may be due to the complex pattern of human migration in Brazil, including inter-state movement for work opportunities and historical waves such as during the slave trade and colonization; all potentially leading to multiple introductions of genetically different *Plasmodium* parasites.^28^, ^29^, ^30^, ^31^

Previous studies have shown that South American *P. vivax* parasites form a distinct global subpopulation,^32^, ^33^, ^34^ with informative barcoding loci found within orthologs of genes known to be important for mosquito development stages and possible targets to inhibit parasite transmission, including the *pvcrmp* gene family (with orthologs in *P. berghei* associated with sporozoite development and onwards transmission^35^^,^^36^), *pvs47* and *pvs48/45*.^37^ Drug susceptibility loci are also informative for barcoding, including *pvmdr1*, whose ortholog in *P. falciparum* is associated with multi drug resistance.^33^^,^^34^^,^^38^^,^^39^ *P. vivax* parasites within South America are known to demonstrate general country level separation,^28^^,^^32^ with Brazilian and Peruvian isolates clustering together.^28^^,^^34^ The *P. vivax* parasite population structure in Brazil remains unclear, with the vast majority of currently available WGS data collected from malaria infections in Acre.^32^^,^^40^^,^^41^ Brazil has a complex setting, due to both the three distinct *P. vivax* transmission foci, and the context of human migration. Here, we perform a population genomic analysis of the largest WGS dataset for *P. vivax* isolates from 10 regions within Brazil (n = 123) spanning 7 states, position them in a global context using a filtered global database (n = 885), characterise the within country and wider regional ancestral and population structure, and identify loci under selective pressure. We reveal a complex population of parasites within Brazil, with vast genomic diversity in areas of high transmission, and Brazilian specific signals of selection in genes associated with drug susceptibility.

## Methods

### Whole genome sequence data

A total of 1113 isolates were analysed, including publicly available (n = 1023)^32^^,^^34^^,^^40^^,^^42^, ^43^, ^44^ and novel sequence data from Brazil (n = 89). After quality control (as described in Bioinformatic analysis), the dataset consisted of 885 isolates spanning all regions where *P. vivax* infections are endemic: (i) South America (n = 315: Brazil 123, Colombia 34, Guyana 3, Mexico 20, Panama 46, Peru 89); (ii) East Africa (n = 84; Eritrea 13, Ethiopia 53, Madagascar 4, Sudan 9, Uganda 5); (iii) South Asia (n = 114; Afghanistan 27, Bangladesh 1, India 48, Pakistan 37, Sri Lanka 1); (iv) South East Asia (SEA; n = 286; Cambodia 71, China 12, Laos 2, Myanmar 28, Thailand 160, Vietnam 13); and (v) the Western Pacific and southern South East Asia (SSEA; n = 86; Indonesia 9, Malaysia 50, Papua New Guinea 26, The Philippines 1) (Table S1). These included newly sequenced isolates (n = 51) and publicly available data (n = 834) in the final filtered dataset. Newly sequenced isolates were obtained from whole blood samples from seven states in Brazil (Acre 4; Amapá, 10; Rondônia, 4; Amazonas 3; São Paulo 12; Mato Grosso 5; Pará 13), leading to a total of 123 high quality WGS data from isolates within Brazil spanning all areas of *P. vivax* transmission (see Fig. S1 for a map; Table S2).

The whole blood samples were obtained from symptomatic malaria patients. All samples were collected with the appropriate ethical approval from relevant authorities, including from Hospital Universitário Antonio Pedro, Universidade Federal Fluminense (ref. CAAE 06214118.2.0000.5243) and Faculdade de Medicina de São José do Rio Preto (ref. CAAE 01774812.2.0000.5415), Centro de Pesquisa em Medicina Tropical Rondônia (CAAE 61442416.7.0000.0011), and Instituto de Ciências Biomédicas (ICB/USP; ref. CAAE: 03930812.8.0000.5467). Informed consent was obtained from all individuals. DNA was extracted from whole blood samples using the QIAamp DNA Blood Mini Kit (Qiagen), quantified using a Qubit (v2.0) fluorometer, and single-species *P. vivax* infections were confirmed using qPCR. Selective whole genome amplification (SWGA) using a set of previously described primers^45^ was used to increase the relative levels of *P. vivax* DNA within the sample, allowing for whole genome sequencing (WGS).^46^ Amplified isolates were sequenced using the Illumina MiSeq and HiSeq4000 platforms using paired-150 bp read kits through The Applied Genomics Centre, LSHTM.

### Bioinformatic analysis

FASTQ files generated from the Illumina sequencing reads (from both publicly available (n = 1023) and newly sequenced isolates (n = 89), available from the European Nucleotide Archive under project codes PRJEB56411, PRJEB44419, PRJEB36199 and the MalariaGEN *P. vivax* Genome Variation project,^40^ were trimmed using TRIMMOMATIC (v0.39) with the following parameters: LEADING:3, TRAILING:3, SLIDINGWINDOW:4-20, MINLEN:36.^47^ Trimmed reads were aligned to the PVP01 *P. vivax* reference genome (v1)^48^ (https://plasmodb.org) using BWA-MEM software (v0.7.12).^49^ BAM files were processed using *samtools* (v1.10) functions fixmate and markdup. We used a “training set” of high-quality *P. vivax* SNPs from previously published work^50^ to calibrate variant calling (see^34^). Using the training set, GATK's BaseRecalibrator and ApplyBQSR functions were run within a robust framework^51^*,* to produce improved and corrected BAM files for all isolates. SNPs and indels were determined using GATK's HaplotypeCaller (v4.1.4.1; options -ERC GVCF; otherwise default settings) to produce variant call format files, which contain all SNPs and insertions and deletions (indels) identified.^51^ The GATK ValidateVariants function was used to validate the resulting Genomic Variant Call Format Files (GVCFs), which were subsequently imported into a GenomicDB using the GATK function GenomicsDBImport. GATK's GenotypeGVCFs function was used to create a combined VCF including all isolates. A total of 3,932,759 unfiltered SNPs were identified across the 1113 isolates. Variants within subtelomeric regions and Variant Quality Score Log-Odds (VQSLOD) scores <0 were removed. A total of 228 isolates with more than 40% of SNPs missing genotype data were excluded from downstream analysis. The final dataset consisted of 885 isolates and 454,681 high quality SNPs used for population genetic analysis. SNPs were annotated with their downstream effect using SnpEff software.^52^

### Population genetic analysis

Multiplicity of infection (MOI) was calculated at a country level using the F_WS_ score implemented in the *moimix* package (https://github.com/bahlolab/moimix), as well as at an individual isolate level using estMOI software.^53^ Population structure of isolates was investigated using a principal component analysis (PCA) based on pairwise SNP Manhattan distances between isolates. Maximum-likelihood (phylogenetic) trees were created using IQTREE software (v2.1.2)^54^ on a nucleotide alignment consisting of the high quality isolates SNP positions. Ancestral analysis was performed using the ADMIXTURE (v1.3.0) package on matrices of high-quality SNPs with a linkage disequilibrium correlation coefficient ≤0.1. ADMIXTURE predicts the most likely number of ancestral populations (K) within a dataset using cross-validation error.^55^ We calculated the (pairwise) fixation indices (*F*_*ST*_) for SNPs between population groups (at global regional, country and two grouping levels within the Brazilian population; clade and geographic groupings) to investigate alleles driving the differences between populations using the VCFtools (v0.1.16) function *--weir-fst-pop*.^56^ Nucleotide diversity (Nei and Li *π*) was calculated genome-wide using VCFtools within each Brazilian state (*Pará*, n = 13; Amapá, n = 10; Mato Grosso, n = 5; Rondônia, n = 5; Acre, n = 74; Amazonas, n = 4; São Paulo, n = 12) using sliding windows of 25 kbp.

### Positive and balancing selection and IBD analysis

We screened monoclonal (F_WS_ >95%) isolates for signals of positive selection at both the regional and country level, with a focus on South American, and specifically Brazilian samples, using the REHH package (v3.2.1) in R.^57^ The integrated haplotype homozygosity score (iHS)^58^ was calculated to identify signals of within population selection, and the Rsb^59^ score was calculated to demonstrate signals of selection between two assigned populations. Both measures were calculated at the regional and country level, as well as within Brazil at two different grouping classifications (clade groupings from the phylogenetic tree, and geographical groupings into Group A and Group B (Table S2)). Candidate regions were identified from iHS and Rsb results using default parameters and a *p*-values of <1 × 10^−4^ and <1 × 10^−5^, respectively. Only populations with >10 isolates and genes with >5 SNPs were included in analysis. Where there were >10 isolates per country, monoclonal Isolates (F_WS_ >95%) were screened at the country level for identity-by-descent (IBD) using the hmmIBD package with default parameters. For IBD analysis, a recombination rate of 13.5 kb per centimorgan (cM) was used, based on previous work in *P. falciparum*^60^ and commonly used in *P. vivax* research.^28^^,^^61^ This *P. falciparum* based recombination rate is the default setting in hmmIBD,^62^ but despite an absence of an equivalent robust estimate for *P. vivax*, genome-wide analysis has shown that the rates may be similar between the two species.^63^ Pairwise comparisons for isolates presenting evidence of IBD were plotted using a sliding window of 50 kbp along the genome location. Signals of selection at the regional level (for populations with >10 isolates), and within Brazil at the gene level (for genes with >5 SNPs), were investigated using the Tajima's D metric, which was calculated using the PEGAS package (v0.14).^64^

### Role of funding source

The funders had no role in study design, data collection and analysis, decision to publish, or preparation of the manuscript.

## Results

### *P. vivax* isolates and sequencing data

WGS data of Brazilian samples (n = 123) includes isolates from human infections spanning 10 regions (Goianésia do Pará, Novo Repartimento, Itaituba (Pará State), Macapá, Oiapoque (Amapá State), Rio Branco (Acre State), Porto Velho (Rondônia State), Barcelos (Amazonas State), and Mato Grosso and São Paulo States), and builds on public sequence data originating from infections in Acre and Rondônia^28^^,^^33^^,^^41^ (see Fig. S1 for a map of all Brazilian isolates). WGS data was analysed with 1113 isolates of *P. vivax* spanning 26 countries, and a total of 3,932,759 SNPs were identified.^32^, ^33^, ^34^^,^^40^ After filtering (see Methods), a final combined “high quality” dataset consisted of 885 isolates with a total of 454,681 unique SNP positions in the core genome of *P. vivax*, excluding the hypervariable regions*.* The filtered isolates were assigned into regional groups: South America (n = 315, including Brazil (n = 123), Colombia, Guyana, Mexico, Panama, Peru), South Asia (n = 114; Afghanistan, Bangladesh, India, Sri Lanka, Pakistan), East Africa (n = 84; Eritrea, Ethiopia, Madagascar, Sudan, Uganda), South East Asia (SEA; n = 286; Cambodia, China, Laos, Myanmar, Thailand, Vietnam), and Southern SEA (SSEA; n = 86; Malaysia, Papua New Guinea, Indonesia, The Philippines). These regions are based on previous genomics work, which demonstrated they are distinct from each other^34^ (Table S1, Table S2, Fig. S1). As expected, the overall sequence coverage before quality control (mean 49-fold; median 16.7-fold) was lower than post-filtering (mean 60.6-fold; median 29.5-fold).

### Four ancestral populations in South America with a distinct Brazilian parasite population

**Fig. 1:**
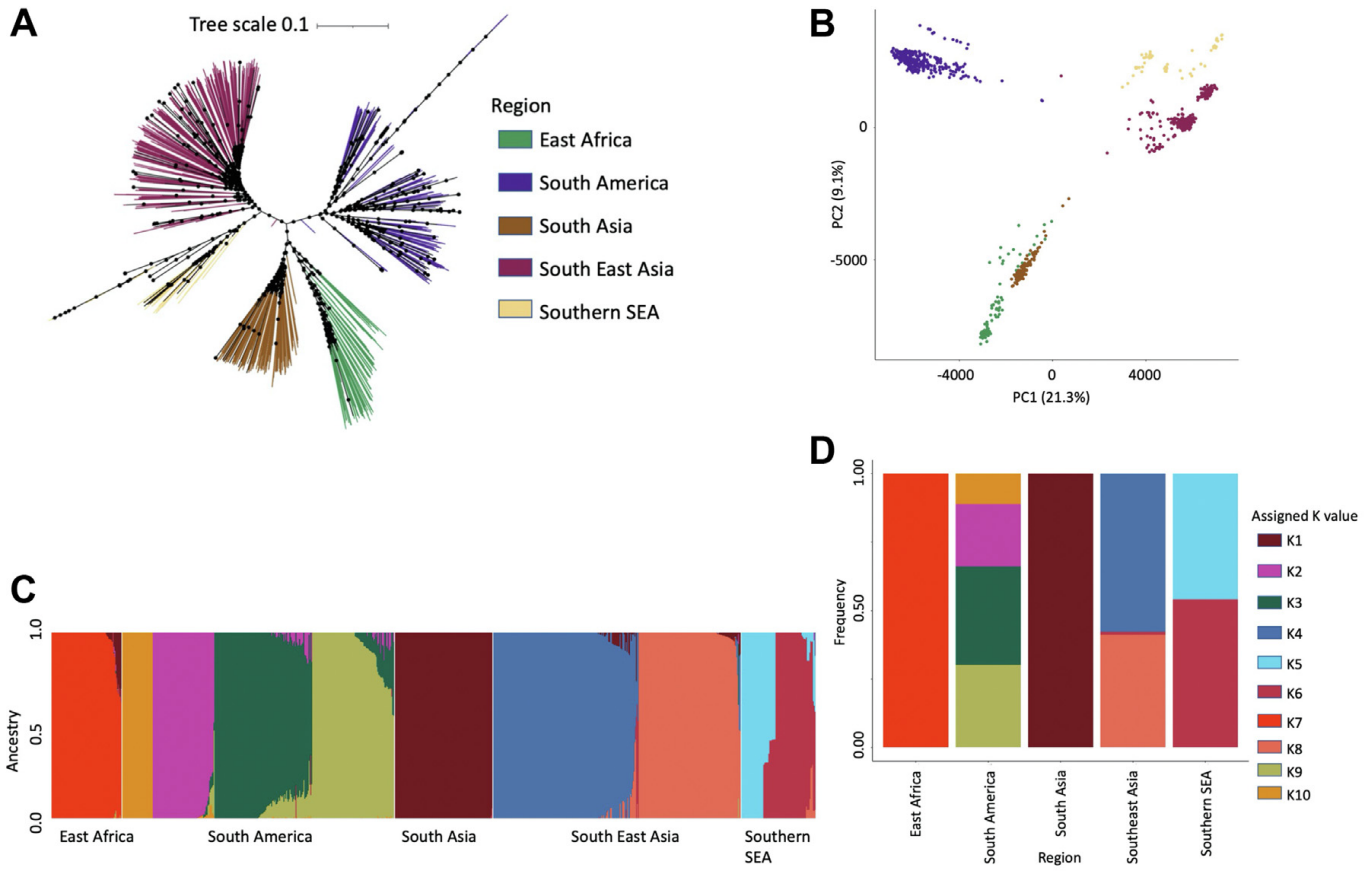
**Population structure of 885 *P. vivax* isolates from 26 countries**. **A)** Maximum likelihood phylogenetic tree generated using IQTREE from the pairwise SNP matrix of the complete global dataset of 885 samples and 454,681 SNPs. IQTREE was run using ModelFinder, tree search, ultrafast bootstrap and SH-aLRT test. Bootstrap scores between 50% and 100% are annotated on the tree branches with a black circle. Branches are colouring according to regional grouping (East Africa, n = 84, green branches; South America, n = 315, purple branches; South Asia, n = 114, brown branches; Southeast Asia (SEA), n = 286, dark pink branches; Southern SEA, n = 86, yellow branches). The phylogenetic tree file was visualised in iTOL with midpoint rooting. **B)** Principal component (PC) analysis displaying principal components 1 and 2 of the distance matrix generated using the SNP matrix. Each point represents and individual sample, coloured according with the region assigned in **(A)**. PC 1 summarises 21.3% of the total variation whilst principal component 2 summarises 9.1% of the total variation. **C)** ADMIXTURE analysis of the global dataset predicted a total of 10 ancestral populations spread across each region: East Africa, n = 1; South America, n = 4; South Asia, n = 1; SEA, n = 3; SSEA, n = 2. **D)** Bar plot summarising the number and proportion of each ancestral population within each region.

**Fig. 2:**
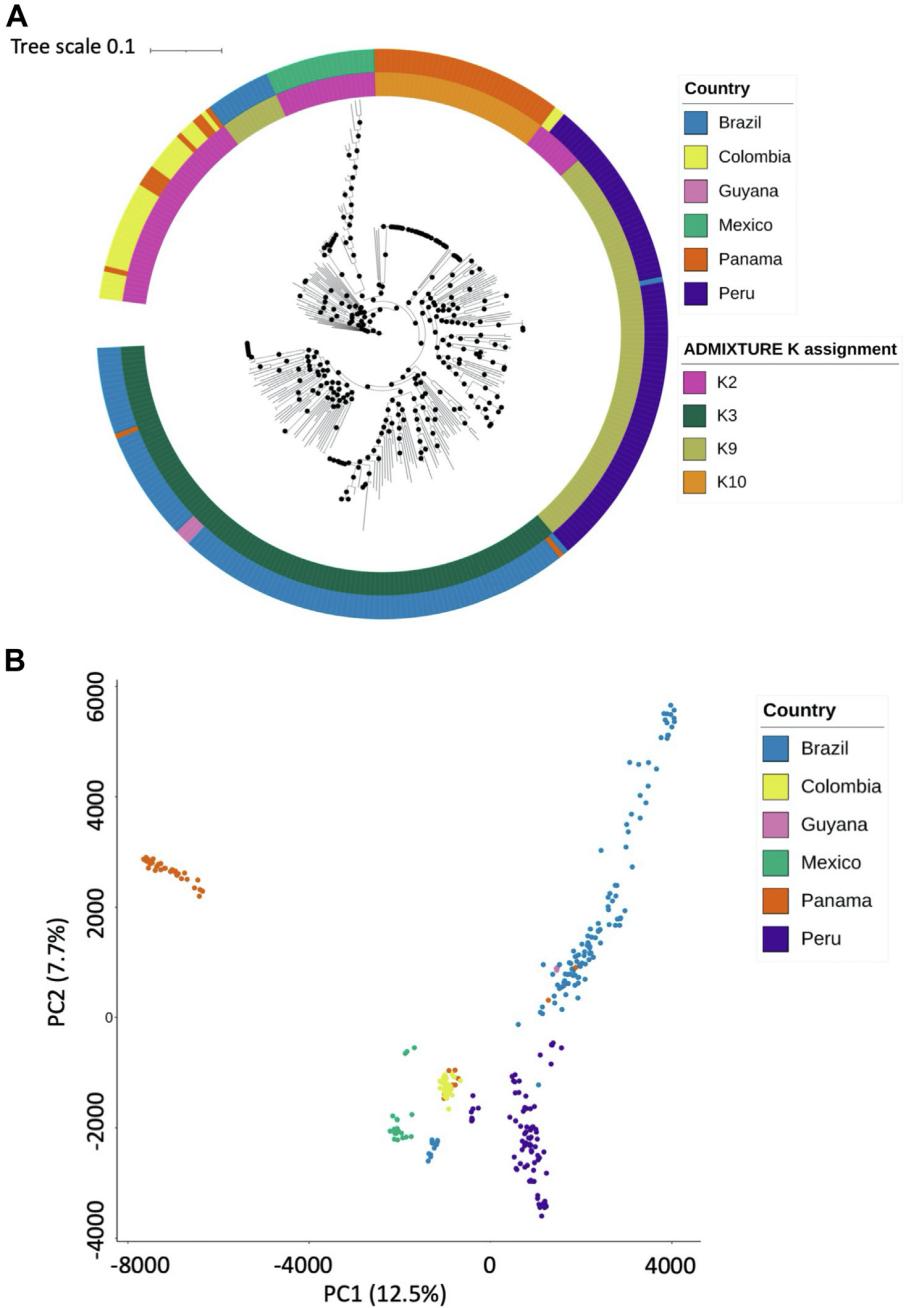
**Population structure of South American isolates**. **A)** Maximum likelihood (ML) tree using SNP data (102,765 unique SNPs) from 315 isolates from South America (Brazil, n = 123; Colombia, n = 34; Mexico, n = 20; Panama, n = 46; Peru, n = 89; Guyana, n = 3). The outer circle track is coloured according to the country of each isolate (Brazil - blue, Colombia - yellow, Guyana - pink, Mexico - green, Panama - orange, Peru - purple), and the inner circle track denotes the population assigned to each isolate after ADMIXTURE analysis of the entire global dataset. ADMIXTURE denoted four populations within South America (K2 - pink, K3 - dark green, K9 - light green, K10 - orange). IQTREE was used to generate ML trees using ModelFinder software (which calculated GTR + F + R10 as the model with the best fit), tree search, ultrafast bootstrap and SH-aLRT test. **B)** Principal component analysis of the pairwise distance matrix generated using the 102,765 SNP matrix from 315 South American isolates. Each point denotes a sample, which is coloured according to the country, as with the tree in **A)**.

**Fig. 3:**
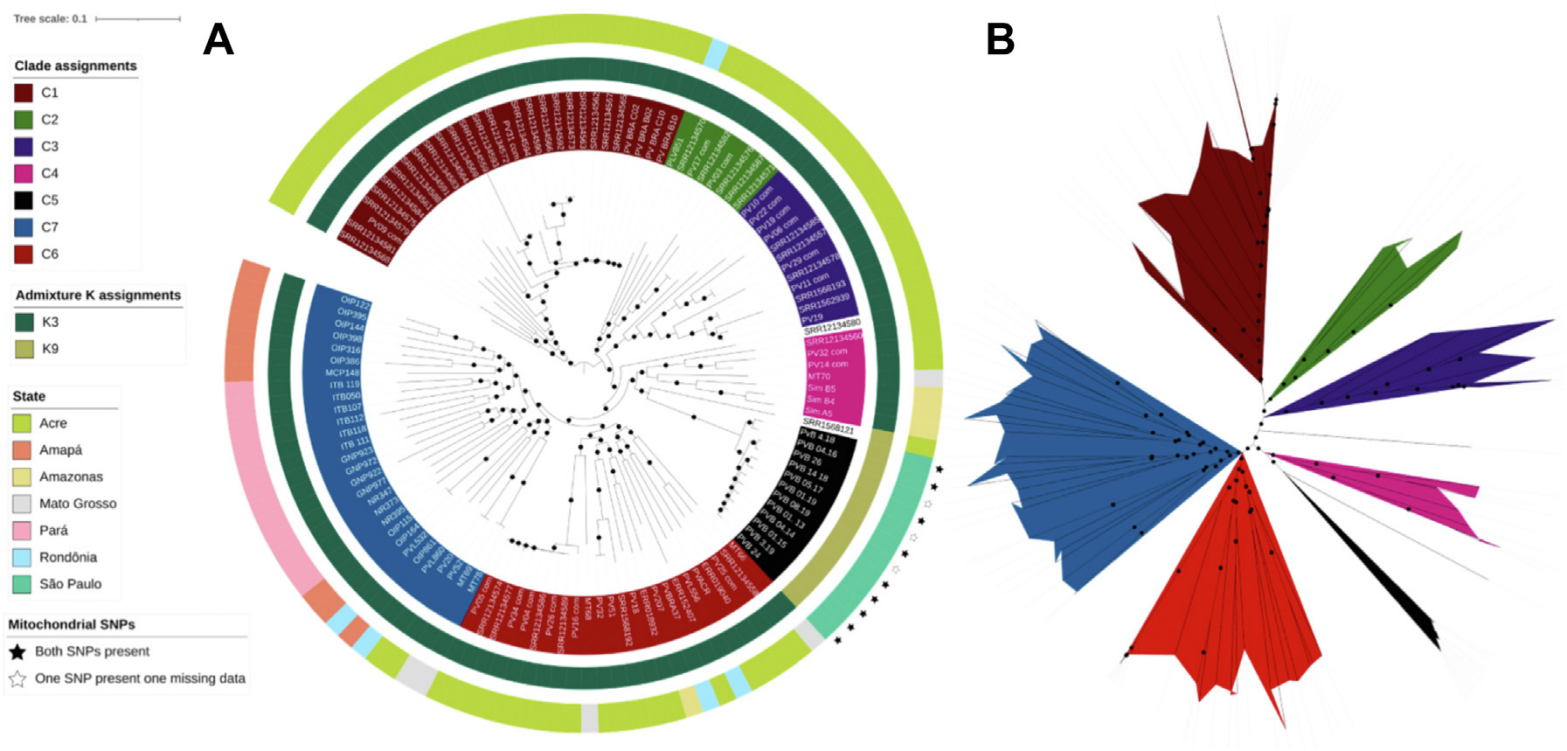
**Population structure within Brazil isolates**. Maximum likelihood tree using SNP data (70,757 unique SNPs) from 123 isolates from seven states within Brazil. **A)** Circular phylogenetic tree in iTOL, with outer colour strip coloured according to the state (Pará, n = 13; Amapá, n = 10; Mato Grosso, n = 5; Rondônia, n = 5; Acre, n = 74; Amazonas, n = 4; São Paulo, n = 12), the inner colour strip highlighting the ADMIXTURE population assignments from the global analysis (K3, n = 13; K9, n = 110), and the inner label coloured according to the 7 clades assigned based on the tree topology (C1, n = 29; C2, n = 8; C3, n = 12; C4, n = 7; C5, n = 12; C6, n = 24; C7, n = 29). Two isolates, SRR12134580 and SRR1568121 were not assigned a clade grouping. Isolates containing the two investigated mitochondrial SNPs (T4133C and A4467G in PvP01_MIT_v1) are labelled using a black star on the outer perimeter of the colour track; isolates containing both SNPs have a black filled in star, isolates where one SNP is present, but there is missing data for the other SNP are denoted with a white star with a black outline. **B)** Mid-point rooted visualisation of the same tree in **A)** to demonstrate clade groupings. The maximum likelihood tree for both plots was generated using IQTREE with ModelFinder software (which assigned TVM + F + R5 as the model with the best fit), tree search, ultrafast bootstrap and SH-aLRT test. Bootstrap values between 50% and 100% are indicated by a black circle midway along each branch length.

Both SNP-based maximum likelihood tree and principal component analysis (PCA) on the final dataset (n = 885 isolates, unique SNPs = 454,681) revealed the expected regional separation^34^ of *P. vivax* parasites with distinct clusters forming for South America, as well as for East Africa, South Asia, SEA, and SSEA (Fig. 1). An ADMIXTURE analysis suggested that there are ten ancestral populations spread across the five global regions, including four within South America (K2, K3, K9 and K10) and six elsewhere (East Africa K7; South Asia K1; SEA K8, K6 and K4; SSEA K6 and K5) (Fig. 1, Fig. S2). Within the South American subset of isolates (n = 315), a maximum-likelihood tree and PCA analysis based on the 102,765 unique SNPs, revealed country-level separation, including for Brazil (except São Paulo samples), with some minor overlap between Panama and Colombia, and both Panama and Guyana with Brazil (Fig. 2). There is a high concordance between ADMIXTURE population and country of origin (K3 Brazil, Guyana; K2 Mexico, Colombia; K9 Peru; K10 Panama; Fig. 2), with highly clonal clusters for Mexico and Panama consistent with previous studies.^32^^,^^65^ The samples from São Paulo (n = 12) cluster together in a clade separated from the remaining Brazilian samples and close to the Mexican clade (Fig. 2 and Fig. 3). These samples could be *P. simium*, being collected in the geographic region where this parasite has been reported, with the majority of them containing two putative *P. simium* barcoding mitochondrial SNPs (T4133C, A4467G).^13^ Deletions in *pvdbp1* and *pvrbp2a* loci reported in *P. simium* but not in *P. vivax*^66^ could not be characterised with high certainty due to poor sequencing coverage at these regions. None of the 12 São Paulo isolates had coverage >5-fold across the length of *pvdbp1, pvrbp2a*, and their wider flanking regions, leading to uncertainty in deletion calling.

### Loci informative for South American and Brazilian population differentiation

The fixation index (*F*_*ST*_) was used to identify genes driving the *P. vivax* population differentiation for South American and its country-wide isolates. The isolates from São Paulo that may be *P. simium* infections were removed from further *P. vivax* population genetic analyses due to inconclusive species identification. When comparing South American isolates (n = 303) to other regions, the greatest number of highly differentiating (*F*_*ST*_ ≥0.99) SNPs are seen with SSEA (>150 SNPs with *F*_*ST*_ ≥0.99) (South Asia 44, Southeast Asia 37, East Africa 22) (Table S3). Across all pairwise regional comparisons, highly differentiating (*F*_*ST*_ ≥ 0.99) SNPs in South America were found in genes potentially involved in gene regulation (*pvjmjc1*^67^), mosquito life stages (*pvcrmp3*, *pvp28*, *pvp47*, *pvp48/45*^36^^,^^68^, ^69^, ^70^), drug resistance (*pvmdr1*^71^), gliding motility and cell traversal (*pvtrap, pvtlp*^72^^,^^73^), and those encoding parasite surface proteins (*pvmsp10*^74^) (Table S3). These genes overlapped with South American-specific differentiating SNPs (*F*_*ST*_ >0.9, vs. non-South American, n = 570; Table S4; Table 1). A nonsynonymous SNP leading to amino acid substitution 698S > 698G in *pvmdr1*, fixed in both the Brazilian and South American population, differentiated South American parasites from those in SSEA and SEA (*F*_*ST*_ = 1 and 0.99, respectively) in accordance with previous findings^34^ (Table 2, Table S3).

**Table 1:** Non-synonymous SNPs with top 20 *F*_*ST*_ scores that differentiate *P. vivax* isolates from South America and Brazil.

Region	Chr	Pos	Ref	Alt	Gene name	AA change^a^	Nucleotide change	Fst^b^
South America	13	337753	A	C	CRMP3	1719K > 1719N	337753A > C	0.999
South America	12	327858	G	A	P48/45	418R > 418K	327858G > A	0.998
South America	11	1265741	A	T	PVP01_1129500	236N > 236F	1265740A > T+1265741A > T	0.998
South America	11	1265741	A	T	PVP01_1129500	236N > 236I	1265741A > T	0.998
South America	12	323603	C	T	P47	24L > 24F	323603C > T	0.996
South America	11	1276259	A	T	PVP01_1129700	2225T > 2225S	1276259A > T	0.993
South America	13	336962	G	A	CRMP3	1456V > 1456M	336962G > A	0.992
South America	12	424886	A	T	PVP01_1210400	195R > 195W	424886A > T	0.990
South America	14	1322275	C	A	PVP01_1430500	1067L > 1067I	1322275C > A	0.986
South America	11	1272806	G	A	PVP01_1129700	1074E > 1074K	1272806G > A	0.986
South America	4	652108	G	C	P230p	158L > 158V	652108G > C	0.985
South America	7	747984	G	A	PVP01_0716800	575G > 575S	747984G > A	0.982
South America	9	1199491	C	T	PVP01_0927300	572E > 572K	1199491C > T	0.980
South America	11	1483830	A	G	PVP01_1134800	579K > 579R	1483830A > G	0.975
South America	14	2662867	T	A	PVP01_1461600	403I > 403L	2662867T > A	0.973
South America	9	892855	G	C	PVP01_0920500	1053Q > 1053H	892855G > C	0.973
South America	9	878674	C	G	PVP01_0920200	517G > 517A	878674C > G	0.967
South America	11	1262951	G	A	PVP01_1129400	20P > 20S	1262951G > A	0.966
South America	8	556253	G	A	PVP01_0813100	150S > 150N	556253G > A	0.966
South America	11	1514101	T	C	ApiAP2	319N > 319D	1514101T > C	0.962
Brazil	10	481636	C	T	MDR1	500D > 500N	481636C > T	0.921
Brazil	12	1621163	C	G	ApiAP2	869R > 869G	1621163C > G	0.895
Brazil	13	818665	T	C	PVP01_1317400	39K > 39E	818665T > C	0.876
Brazil	13	809067	G	A	PVP01_1317200	1086R > 1086Q	809067G > A	0.876
Brazil	5	440493	T	C	NT2	117F > 117S	440493T > C	0.875
Brazil	2	377716	C	A	PVP01_0209100	590G > 590V	377716C > A	0.869
Brazil	12	1618925	A	G	ApiAP2	123I > 123V	1618925A > G	0.868
Brazil	4	530215	T	C	PVP01_0412900	299E > 299G	530215T > C	0.860
Brazil	1	716831	A	T	PVP01_0116000	4344L > 4344M	716831A > T	0.859
Brazil	12	1860075	C	T	PVP01_1245000	1553A > 1553T	1860075C > T	0.853
Brazil	13	810706	G	A	PVP01_1317200	1578G > 1578D	810706G > A	0.849
Brazil	11	915559	G	T	PK4	1694T > 1694N	915559G > T	0.839
Brazil	6	179243	A	T	PVP01_0604500	441L > 441M	179243A > T	0.835
Brazil	9	1366817	C	G	SR1	236E > 236Q	1366817C > G	0.832
Brazil	14	2887017	C	T	PVP01_1467700	33A > 33T	2887017C > T	0.830
Brazil	14	2153846	G	T	PVP01_1449600	1581P > 1581T	2153846G > T	0.820
Brazil	10	490615	C	G	PVP01_1011000	842G > 842A	490615C > G	0.818
Brazil	14	115657	A	G	RBP2a	719K > 719E	115657A > G	0.815
Brazil	13	336738	C	T	CRMP3	1381P > 1381L	336738C > T	0.815
Brazil	7	360367	A	C	PVP01_0706700	544K > 544Q	360367A > C	0.815

a AA amino acid.

b Within Region vs. all other isolates.

**Table 2:**
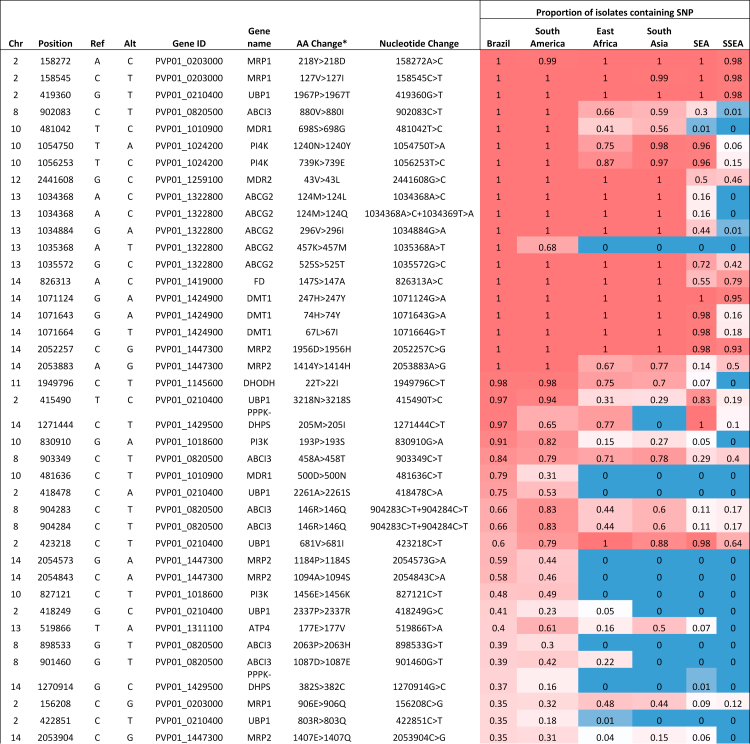

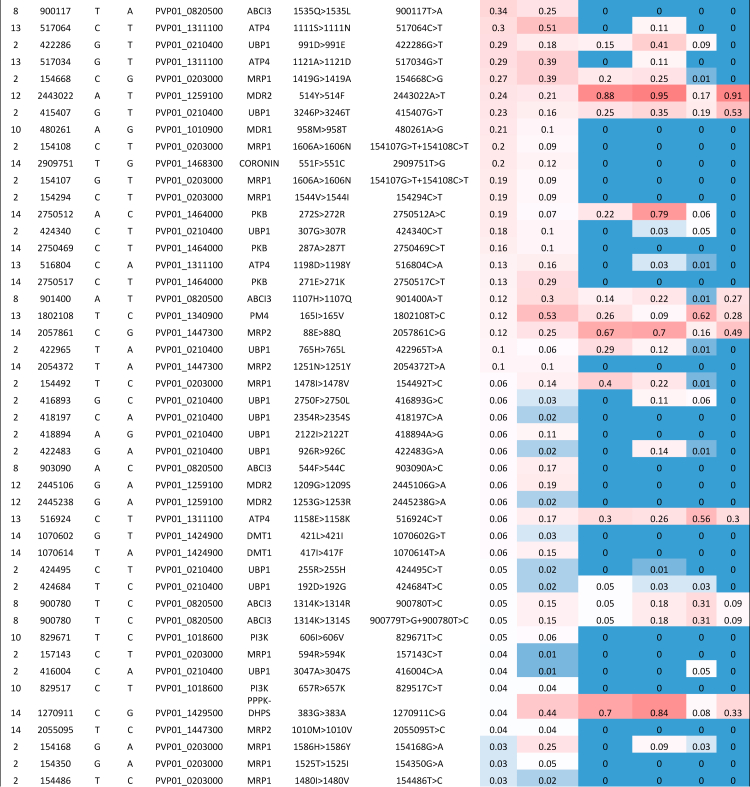

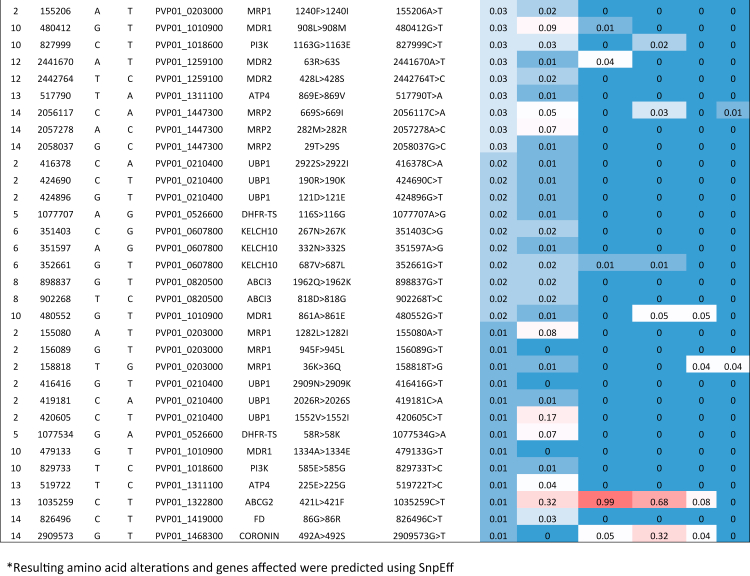
Mutations in putative drug resistance genes in Brazil, with reference to other regions across the globe.

Frequencies from 0 (blue) to 1 (red).

SEA, Southeast Asia; SSEA, Southern Southeast Asia.

^a^Resulting amino acid alterations and genes affected were predicted using SnpEff software.

Within South America, Brazilian *P. vivax* (n = 111, vs. other South America, n = 192) informative SNPs (F_ST_>0.8) were found within genes associated with drug susceptibility (*pvmdr1*^71^), gene expression (*pvapiap2*^75^), mosquito life stages (*pvcrmp3*^36^) and a gene encoding reticulocyte binding protein, *pvrbp2a*^76^ (position 719K > 719E, *F*_*ST*_ = 0.82) (Table 1, Table S5). By focusing on country-level pairwise comparisons with Brazil, the highest number of differentiating SNPs (*F*_*ST*_ >0.95) were observed against Mexico (62 SNPs), followed by Panama (29 SNPs), Colombia (14 SNPs) and Peru (4 SNPs) (Table S6), consistent with differences in geographical distance and genetic clustering in the maximum likelihood tree and PCA analysis (Fig. 2). Of note, are putative drug resistance mutations, including within *pvpppk-dhps* (M205I: Brazil 97% vs. Mexico 0%; *F*_*ST*_ = 0.95; S. America 65%) previously observed in China^77^ and Thailand,^78^ and *pvmdr1* (V221L: Panama 92% vs. Brazil 0%; *F*_*ST*_ = 0.97) previously observed in Peru^79^ (Table S6).

### Distinct populations within Brazil associated with parasite surface proteins and drug resistance loci

All Brazilian regions have similarly low levels of nucleotide diversity (average *π* across all states, excluding São Paulo, 3.06 x 10–4), with the lowest diversity seen within São Paulo (*π* = 0.54 x 10^−4^) (Fig. S3). A SNP-based maximum likelihood tree (n = 70,757 SNPs) of only Brazilian isolates (n*=* 123) revealed seven distinct clades (C1–C7) (Fig. 3), including a likely *P. simium* clade with the samples from São Paulo (C5), corresponding to the ADMIXTURE population K9 (Fig. 3, Fig. S4). Clades C1, C2, C3, C4 and C6 mostly cover isolates from Acre and the Amazonas, with a small number of isolates from Rondônia in clades C2 (n = 1) and C6 (n = 2), and a small number of isolates from Mato Grosso in clades C4 (n = 1) and C6 (n = 2), demonstrating the vast genetic variability of isolates in the Amazon basin. Clade C7 covers isolates from Amapá and Pará located in northern Brazil, with a small number of isolates from Rondônia (n = 2), Acre (n = 2), and Mato Grosso (n = 2) (Fig. 3, Fig. S4). Two isolates from Acre did not fall into a clade grouping (Fig. 3). Whilst the population structure within Brazil appears to be complex, it is important to note that excluding those from São Paulo, all other Brazilian isolates clustered together as population K3 in the global ADMIXTURE analysis, which was a distinct population of Brazilian isolates (Fig. 3).

Informed by the population structure observed, subsequent analysis within Brazil compared different clades as well as two regional groups (A: Amazonas, Acre, Mato Grosso and Rondônia states (n = 88); B: Amapá and Pará states (n = 23)) (Fig. S5, Table S2). *F_ST_* scores are heavily impacted by population size, therefore only clades with >15 isolates were compared to each other (excluding clades C2 to C4 from comparisons) (Table S7). Highly differentiating non-synonymous SNPs (*F*_*ST*_ >0.85) separating clades C1, C6 and C7 were identified (Table S7), including in many conserved proteins of unknown function and genes associated with reticulocyte binding (merozoite surface protein, *pvmsp1*),^80^ liver stages of infection (*pvlisp2*^81^), and within a Plasmodium*-*specific ABC transporter (*pvabci3)* whose ortholog has been linked to a drug resistance mechanism in *P. falciparum*.^82^ Clades C6 and C7, which are associated with isolates from Acre and Amapá-Pará states respectively only have 11 highly differentiating mutations (*F*_*ST*_ >0.85), all synonymous SNPs. When comparing regional groups A and B, most highly differentiating SNPs were observed on chromosome 6 within the Plasmodium interspersed repeat gene family (*pvpir*). *Pvpir* genes are the largest gene family within Plasmodium spp (found within *P. vivax,* as well as simian and rodent malaria parasites), thought to play a role in host red blood cell invasion and immune evasion^83^ (Table S8).

### Multi-clonality and signals of relatedness and homology within parasite populations

**Fig. 4:**
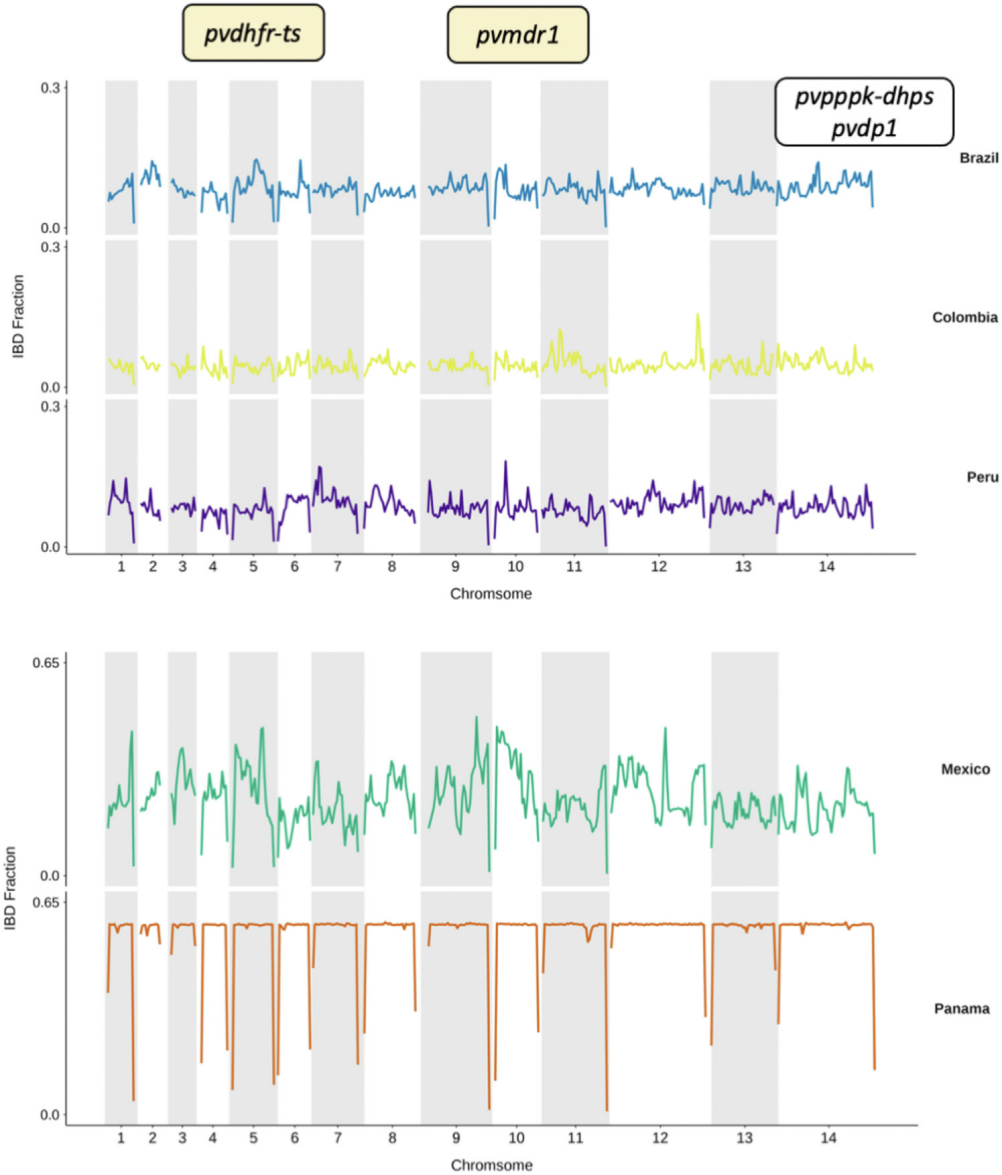
**Country level comparisons of identity by descent (IBD) across the whole genome of monoclonal *P. vivax* isolates.** IBD fractions along 50 kbp sliding windows across the genome at country level separation. The top 1% of IBD fractions for each country is summarised in Table S10. Genes of interest which demonstrate high signals of IBD are annotated. Where signals of high IBD are conserved across all countries within South America, the gene annotation is at the top of all plots and highlighted in yellow (*pvdhfr-ts* on chromosome 5 and *pvmdr1* on chromosome 10). For country specific signals of high IBD where genes of interest are found, the gene annotation is found above the line graph for each country (*pvpppk-dhps/pvdbp1* within chromosome 14 in Brazil).

Multi-clonality, as measured by within-sample diversity (F_WS_ metric < 95%), was present in 206 (23.2%) of all isolates, being more common among SEA (35.4%) and SSEA (33.7%), suggesting a higher chance of co-transmission of multiple *P. vivax* strains in these regions (Table S1, Fig. S6). In the Brazilian isolates, monoclonal infections were common, with F_WS_ >0.95 observed in 87.8% of the 123 isolates. Multiclonality was more common within clades C4, C6 and C7 (28.6%, 20.8% and 24.1%, respectively, of all isolates with F_WS_ <0.95). Multiclonality was also more likely in regional group B (30.4% of all isolates with F_WS_ <0.95) than group A (9.1% isolates F_WS_ <0.95) (Table S2, Fig. S7). Multiclonality appears less common in this analysis than previously presented for isolates from the region of Mancio Lima,^44^ which is likely due to differences in SNP filtering, where we perform F_WS_ analysis on the already filtered dataset. Analysis of identity-by-descent (IBD), to quantify isolate relatedness, was performed at country level on the global dataset of monoclonal isolates (n = 679) (Table S1), and revealed Malaysia (median IBD 0.335), Panama (0.971) and Mexico (0.232) with the greatest fractions of IBD, with all other populations with fractions less than 0.0561 (Ethiopia 0.0561, Peru 0.0544, Colombia 0.0462, Brazil 0.0426, India 0.0236, Pakistan 0.0137, Cambodia 0.0123, Afghanistan 0.0121, Myanmar 0.00698, Papua New Guinea 0.00607) (Fig. S8, Table S9). Across genome-wide sliding windows of 50kbp, there are several global patterns of signals of high IBD (Table S10). In Brazil, a segment on chromosome 5 encompassing *pvdhfr-ts*, a gene associated with pyrimethamine resistance, demonstrates a high signal of IBD (0.122) (Fig. 4).^39^ Additionally, there is a segment of chromosome 10 encompassing *pvmdr1*, a gene associated with multi-drug resistance (Brazilian IBD = 0.136), which demonstrates a high signal also in East Africa (0.124) and South America (0.276)^39^ (Fig. 4). Brazil also has a high signal of IBD on chromosome 14, observed in other countries, where both *pvdbp1*, a gene associated with erythrocyte invasion,^84^ and *pvdhps-pppk,* a gene associated with sulfadoxine resistance,^39^ are found (Brazilian IBD = 0.133) (Fig. 4).

We investigated patterns of IBD for clades C1, C6 and C7 (all with >15 isolates). Clade C1 specific signals of IBD were found within chromosomes 9 (encompassing *pvama1*, a potential vaccine candidate^85^) (IBD = 0.692), and 3 sequential segments within chromosome 14 (positions 2.35Mbp to 2.50Mbp), which included many genes, some of unknown function (Table S11**)**. In this region a gene encoding the clustered-asparagine-repeat-protein (*pvCARP*) is also found, which is associated with the host immune response to malaria infection.^86^ For clade C6, signals were identified on chromosome 3, 5, 11, 12, 13 and 14 (IBD >0.3), encompassing a GPI-anchored micronemal antigen (*pvGAMA*) on chromosome 5 which is an essential invasion protein in *P. falciparum* infections, suggested as a potential vaccine candidate,^87^ and loci encoding putative AP2 domain transcription factors associated with gene regulation.^75^ Clade 7 IBD signals were found in chromosome 14, where both *pvdbp1*, a gene associated with *P. vivax* erythrocyte invasion,^84^ and *pvpppk-dhps,* a gene associated with sulfadoxine susceptibility,^39^ are located (IBD = 0.134) (Table S11). High signals of IBD were observed across all three clades (C1, C6 and C7) within chromosome 14 (average IBD = 0.329). Signals of IBD across the two geographical groupings (A, B) were polarizing, with signals in chromosomes 2 and 5 for Group A, including a region encompassing the eukaryotic initiation factor-2α, potentially associated with artemisinin resistance in Plasmodium parasites.^88^ For Group B, there were within segments of chromosome 14, including among other genes, the *pvpppk-dhps, pvdbp1* and *pvrbp1a*, a gene associated with erythrocyte invasion^89^ (Table S12).

### Regions under selection in South American and Brazilian subpopulations

**Fig. 5:**
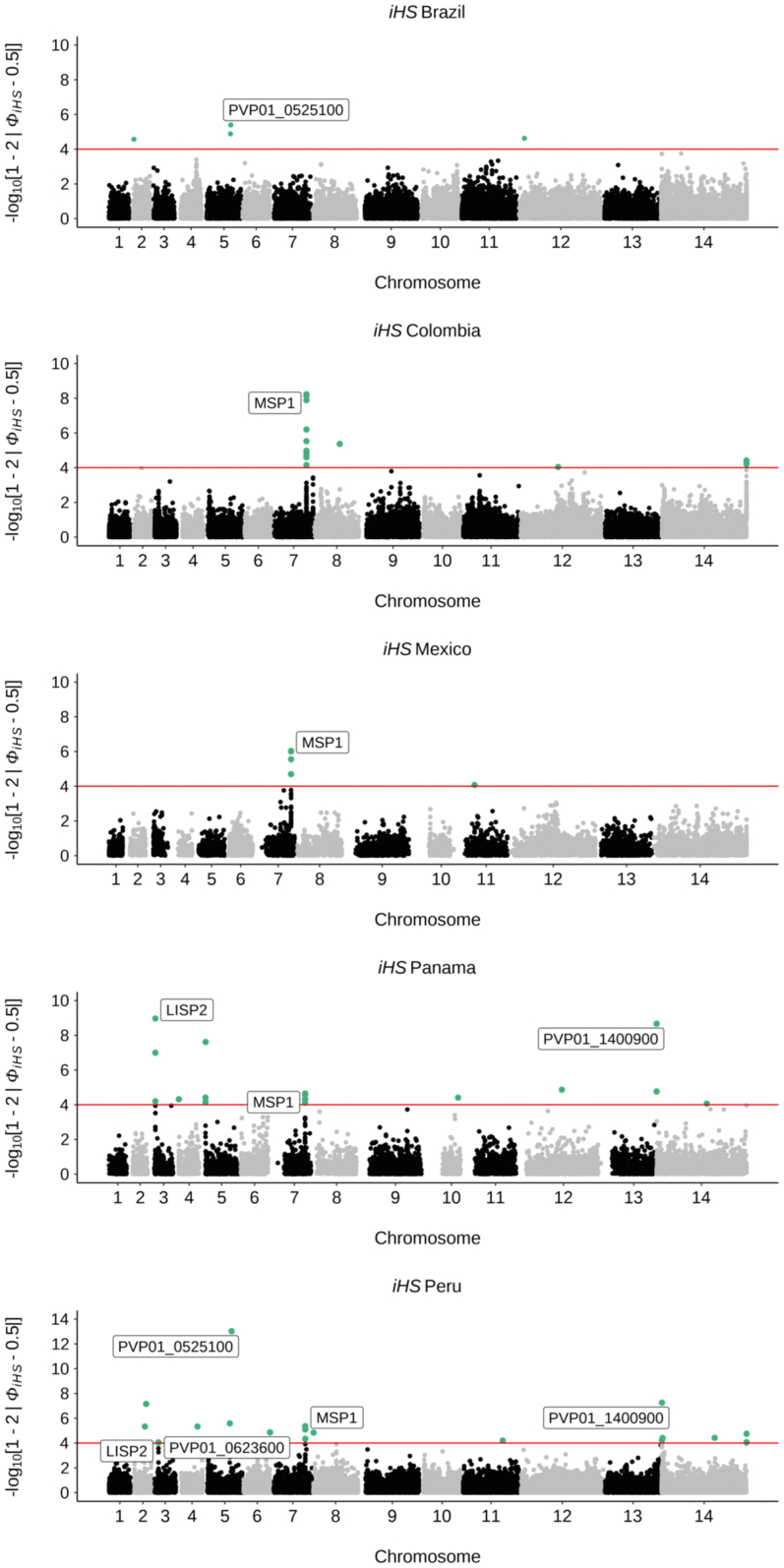
**Evidence of selection (iHS) within South American countries**. Genome-wide iHS scores in a Manhattan plot for all countries within South America where there are >10 isolates. SNPs within genes with iHS score of P< 1 × 10^−4^ are highlighted in green and gene names are annotated for candidate regions of high iHS for genes with validated functions (MSP1, PVP01_0728900; LISP2, PVP01_0304700). For expanded gene families and genes with unknown functions, gene IDs are given (PVP01_0525100, PHIST protein; PVP01_1400900, exported plasmodium protein of unknown function (PUF); PVP01_0623600, PIR protein. Raw outputs of iHS scores, alongside proposed candidate regions for South America and Brazil specifically are summarised in Tables S14–S16.

Genome-wide analysis to identify positive selective sweeps was performed using the “single population” integrated haplotype score (iHS) across monoclonal isolates (n = 679). Surface protein genes (*pvmsp1, pvmsp4, pvmsp5*) were detected in all global regions except for SSEA (Table S13). Within East Africa, South Asia and SEA, signals of positive selection were identified within chromosome 2 in a region that encompasses several genes including the *pvmrp1*, a gene associated with drug susceptibility.^34^ In both South Asia and SEA, signals of positive selection were found within chromosome 5, where the *pvdhfr-ts* gene is located. Loci associated with erythrocyte binding were also identified, including *pvdbp1* in SEA (Table S13, Fig. S9). Across East Africa, South Asia, SEA, and SSEA, analyses detected signals of positive selection within chromosome 3 which includes *pvlisp2,* linked to parasite development in the liver.^81^ Signals of positive selection within South America include regions of the genome where multiple Plasmodium Poly-Helical Interspersed Sub-Telomeric (PHIST) proteins are encoded on chromosome 5. These proteins peripherally-localised in infected erythrocytes and in *P. falciparum* are involved in functions such as protein trafficking, membrane rigidity and intercellular signalling.^90^ Other loci identified included the leucine-rich repeat protein (*pvlrr8*) and the surface protein *pvmsp1* along with a paralog *pvmsp1p-19*^91^ (Table S13)*.* Within South America, we looked for signals of positive selection at the country level (for countries with >10 isolates). Signals were detected in *pvmsp1* within Colombia, Panama, and Peru, in *pvdbp1* within Peru, and in *pvlisp2* within both Panama and Peru (Fig. 5, Table S14). There were only 5 SNPs detected within Brazil which demonstrated signals of positive selection, with just 2 SNPs in coding regions (Plasmodium exported protein PVP01_0525100, *pvphist*) (Fig. 5, Table S15). Within Brazil, signals of positive selection using iHS were detected in chromosomes 8 and 14 in Amazonian clade C6, including the ABC-transporter *pvabci3* (PVP01_082050), whose orthologue is associated with drug resistance in *P. falciparum*.^92^ In Clade C7 isolates (associated with Amapá and *Pará st*ates) candidate regions for positive selection were seen within loci encompassing surface proteins (e.g., *pvmsp1*, *pvmsp4, pvmsp5)*, *pvlisp2, pvlrr8* and *pvdbp* involved in erythrocyte invasion (Table S16).

**Fig. 6:**
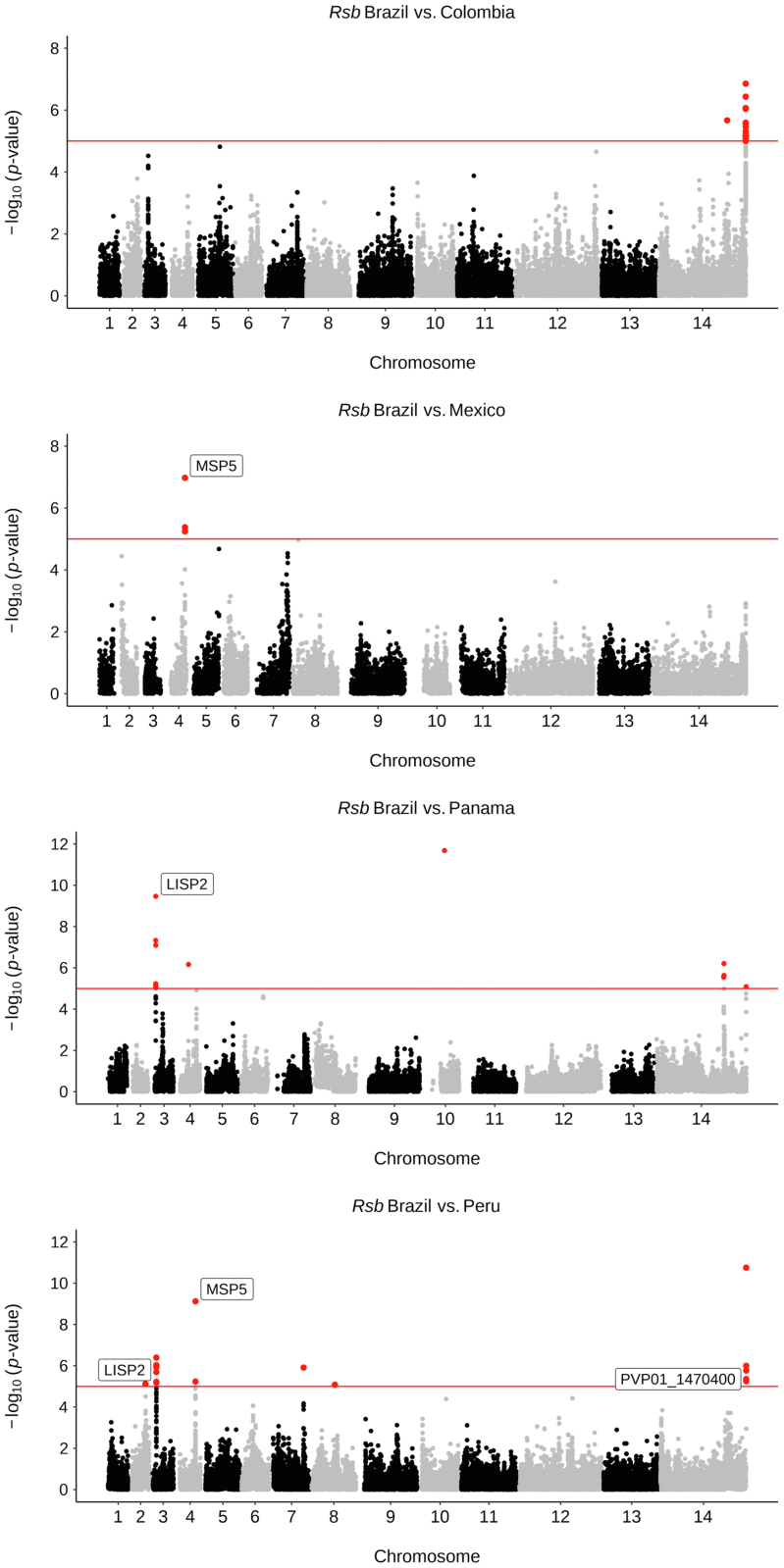
**Evidence of selection between countries in South America (Rsb)**. Manhattan plots for genome-wide Rsb analysis for *P. vivax* isolates within South America at the country level. SNPs with P < 1 × 10^−5^ are highlighted in red, and gene names are annotated for candidate regions of selection. Gene names are given for genes with validated locations and functions (MSP5, PVP01_0418400; LISP2, PVP01_0304700), whereas gene IDs are given for genes within expanded gene families or genes with unknown functions (PVP01_1470400, exported PUF). Rsb results output for South America, in addition to within Brazil analyses are summarised in Tables S17–S20.

The between population Rsb method was applied to detect positive selection at both the regional and country level (Table S17; Table S18; P < 1 × 10^−5^). When comparing South America against the other global regions, multiple SNPs within *pvmsp1*, associated with reticulocyte binding,^80^ demonstrated signs of positive selection. Similarly, SNPs in the gene encoding PvPHIST exported protein were also detected in all pairwise comparisons, except with SSEA. The surface protein encoding gene *pvmsp5* (chromosome 4) was detected between South America and SEA. Comparisons of Brazil to other South American countries revealed multiple SNPs within *pvlisp2* (Brazil vs. Panama, Peru), *pvmsp5* (Brazil vs. Mexico, Peru) and *pvmsp1, pvmsp4* and *pvmsp5* (Brazil vs. Peru) (Fig. 6, Table S18, Table S19). Within Brazil, two candidate genomic regions were detected between clades C1 and C6, where surface proteins were found (e.g., *pvmsp4, pvmsp5*), in addition to two regions between clades C1 and C7 (including *pvsmp1, pvmsp1p,* and *pvlrr8*). Five candidate genetic regions were identified when comparing clades C6 and C7, which included the *pvlisp2* gene and multiple merozoite surface proteins. Between regional groups A and B, 5 candidate regions were identified, which included the *pvlisp2, pvdbp* and *pvmsp1* genes (Table S20).

In addition to positive selection signals, we investigated genes (with >5 SNPs) under balancing selection by applying the Tajima's D statistic to all monoclonal isolates (n = 679). As expected, most Tajima's D values for genes across global regions were negative (median: South America −0.437, SEA -1.82, South Asia −0.756, East Africa −0.385, SSEA -0.904), with the most negative value globally occurring in SEA, suggesting population expansion in this region (Fig. S10). Within South America, median values for Tajima's D were negative in Brazil (−0.034), Colombia (−0.046) and Panama (−0.330), while positive in Mexico (0.173) and Peru (0.078) indicating a population decrease or a genetic bottleneck (Fig. S11). The top 50 genes with the highest and lowest Tajima's D metric in South America are summarised (Table S21)**,** with the most positive including *pvmsp1, pvmsp5, pvlisp2, pvrbp1a,* and many genes encoding exported proteins, including PvPHIST, suggesting balancing selection. The findings from the same analysis for Brazil overlapped, and includes genes *pvmsp1, pvlisp2, pvrbp1a,* and *pvcyrpa*, in addition to loci encoding PvPHIST and PvPIR proteins (Table S22).

### Identification of mutations and allele frequencies in *P. vivax* drug resistance candidate genes

Treatment failures have been reported with *P. vivax* infections, however the molecular determinants for reduced drug efficacy are not clearly defined. We investigated the presence of SNPs within orthologs of genes associated with resistance in *P. falciparum,* alongside loci identified by selection metrics, including hits from previous population genomics studies^34^^,^^40^^,^^50^ (Table 2, Table S23). There are similar patterns of frequencies of SNPs within potential resistance-associated genes between Brazil and the other South American isolates likely due to similar drug regimens across this region. Of note are SNPs which appear close to fixation within the Brazilian population, found within *pvubp1* (potentially associated with artemisinin resistance in *P. falciparum*^93^)*,* multidrug resistance associated proteins MDR1, MDR2, MRP2*,* phosphatidylinositol 4-kinase *pvpi4k* (the target of novel antimalarial class imidazopyrazines^94^)*, DHODH* (a drug target for DSM265, a novel antimalarial in clinical trials, shown to be less effective against *P. vivax* infections than *P. falciparum*^95^)*, ferredoxin – pvfdx* (potentially associated with artemisinin resistance in *P. falciparum*^96^)*, pvpppk-dhps* (associated with sulfadoxine resistance^39^)*,* and genes coding for putative ABC transporters (*pvabci3, pvabcg2*), whose orthologues may be associated with antimalarial resistance in *P. falciparum* infections^71^^,^^92^ (Table 2). Some of these mutations are observed in high frequency in South America but have quite different frequencies compared with other global regions, including a missense mutation within pvmdr1 (698S > 689G), which is fixed in all South American isolates, and found in approximately half of the populations in East Africa and South Asia, but rare and non-existent in SEA and SSEA respectively. Another pvmdr1 mutation (500D > 500N) is also present in Brazil with high frequency (80%) but with lower frequency in wider South America (31%) and not identified in any other continents. Similarly, a missense mutation within pvabcg2 (457K > 457M) is fixed within Brazil and present in South America (69%), but not observed elsewhere (Table 2). Pvabcg2 encodes an ATP binding cassette (ABC) transporter, which are commonly known to be associated with multiple drug resistance phenotypes in many organisms 74 and is linked to the gametocyte stages of parasite development.^97^

## Discussion

Whilst *P. vivax* infections pose a serious risk to global health, genomic analyses of this species, particularly in South America where the parasite is predominant, are scarce in comparison to the more pathogenic *P. falciparum*. Brazil is a unique setting for malaria transmission, with distinct foci relating to the local environments and resultant vector landscapes. To date, all previously published WGS data from Brazil has originated from isolates obtained mainly from Acre and a few from Rondônia, in the north-western region of the country. Previous population genomic analyses have demonstrated that South American isolates (n = 146) are a distinct population with high genetic diversity,^28^ with three ancestral populations (Mexico, Peru, Colombia/Brazil),^34^ in contrast to our study, which reveals four main populations (n = 315; Brazil, Mexico/Colombia, Peru, Panama). Previous work has revealed geographical clustering of isolates from Brazil and Peru,^28^ but whilst closely related in our analysis, they are distinct. Earlier work focused solely on Mancio Lima, and found high levels of inbreeding.^44^ Studies of *P. vivax* from 4 countries (Brazil, Colombia, PNG, India), using microsatellite markers, have demonstrated high similarity between isolates from Brazil (Manaus) and India (Bikaner), and high genetic diversity irrespective of the transmission situation.^98^ Microsatellite data has also shown high diversity within and between Amazon parasite populations (Manaus, Porto Velho), with Amapa and Para infections being the most divergent,^99^ consistent with our findings that also suggest these two states are a distinct diverged clade.

Here, we provide the first insight into the genomic diversity of *P. vivax* isolates from all three malaria endemic regions in Brazil, spanning seven states, to determine the broader population structure within the country, as well as its position within a continental and global resolution. Using 855 global isolates of *P. vivax* across 26 countries, we placed South America in the global context, demonstrating that they form a distinct population with more ancestral populations than other global regions. The four distinct ancestral South American populations mostly correspond to country groups, in accordance with previous studies demonstrating nation-level separation within this continent.^28^^,^^34^ Using 123 isolates from Brazil, we demonstrated that the population structure is complex, with samples clustering across seven distinct clades, clearly separating the Northern states (Amapá and *Pará*) and the highly clonal potential *P. simium* cluster from São Paulo. Isolates from the Amazonian basin fall within five (of the seven) clades, consistent with the high malaria transmission in the large region leading to greater population diversity.

WGS data can reveal genetic differences within and between populations, which may be indicative of signals of differential selection, including those resulting from differences in the implementation of antimalarial drug regimens. For example, artemisinin resistance in *P. falciparum* isolates was confirmed through detecting signals of selection between populations around the *Pfkelch13* gene, agnostic to a resistance phenotype, and mutations in that locus were found to correlate with differences in parasite clearance rates after treatment with artemisinin.^100^^,^^101^ It is therefore possible that genome-wide screens of selection for *P. vivax* may reveal much needed novel candidates of drug resistance. In this context, the monitoring for signals of selection may inform on the effectiveness of control measures, but can also reveal important insights into patterns of parasite adaptation. Understanding the genetic differences across parasite populations can inform on the origin of parasites, leading to the development of molecular barcodes for both *P. falciparum*^102^ and *P. vivax* parasites^33^ to accurately predict the source of infections, including importation events. These geographically informative molecular barcodes can be used as an easier alternative to WGS for determining patterns of parasite transmission, and predicting the source of infection outbreaks, which can be extremely useful in countries nearing elimination to determine between native transmission and imported malaria.

Using comparative population genomics, our results highlight many South American-specific SNPs within genes involved in different parasite life stages and associated with drug resistance. Genes involved in mosquito life stages, such as gametocyte proteins *PVS48/45* and *PVS47*, may be reflective of the different mosquito vectors present in South America compared to other regions, and could be potential molecular barcode candidates for identifying parasites originating in South America. Other studies have also identified mosquito-related proteins under selection in other *P. vivax* endemic regions.^34^^,^^40^^,^^50^ Additionally, South American-specific SNPs were also found within genes encoding parasite surface proteins (e.g., *pvmsp1/4/5*) and drug resistance (e.g., *pvmdr1*). Several signals of selection and homology were identified in loci associated with drug resistance, specifically within *pvdhfr-ts* and *pvmdr1* across all South American isolates, which may reflect similar selection pressures due to a like drug regimens within continent. In addition, a Brazilian-specific signal was observed within *pvdhps-pppk*, the determinant of sulfadoxine resistance in *P. falciparum*. Sulfadoxine resistance in *P. vivax* has been reported in South America but, in contrast to *P. falciparum*, the molecular marker has not been confirmed, in part due to the lack of an *in vitro* culture method for *P. vivax.* Other possible candidates for further investigation linked to antimalarial drugs included *pvmrp1, pvmrp2*, and an ABC transporter I family member (*pvabci3*), revealed as signals of positive selection and/or SNPs fixed in Brazilian samples. The orthologous *pfmrp1* gene in *P. falciparum* is a multidrug-resistance candidate, and has been shown to be under strong selection in across populations, with mutations associated with reduced susceptibility to sulfadoxine-pyrimethamine, chloroquine and mefloquine, and pyronaridine.^103^ The *ABCI3* protein is a Plasmodium-specific ABC family member, and SNP and gene amplification variants in *P. falciparum* have recently been shown to confer anti-plasmodial drug resistance across a variety of compounds.^92^ However, no such investigations of *pvabci3* have been applied to *P. vivax*.

Determining the downstream effect of SNPs for *P. vivax* research is complicated due to the lack of a routine *in vitro* culture method for this parasite species. It is possible to perform orthologue replacement transgenesis in *P. knowlesi*^104^ as this parasite can be cultured in human erythrocytes^105^ and is the most closely related species to *P. vivax*. This system allows the functional investigation of the role of genetic variants, such as in drug susceptibility or red blood cell invasion. Brazil-specific SNPs in genes involved in red blood cell invasion (*pvrbp2a*, *pvrbp1, pvcyrpa*)*,* and signals of positive selection in PHIST family members were also detected, which may reflect immune selective pressure or regional-specific host factors on erythrocytes. Invasion genes are possible *P. vivax* vaccine candidates, and understanding the genetic diversity of these loci across global populations can inform on their potential efficacy.

The liver stage *pvlisp2* gene, which differentiates between dormant hypnozoites and early developing parasites,^81^ was identified when investigating signals of selection across South American populations and within Brazilian clades. Genetic markers in *pvlisp2* can assist the development of drug discovery assays predictive of anti-relapse activity.^81^

Overall, our work provides insights into the genomic diversity across all three malaria endemic regions in Brazil, as well as in the broader context of South America and other continents. The results highlight many novel and previously detected genes and mutations, which may reflect ongoing evolutionary interactions with the vector and human hosts in the different regional settings and in response to antimalarial drugs. Our insights will inform functional studies, which can determine the role of the candidate loci during the parasite life cycle and in response to treatment and anti-relapse therapies. Ultimately, this work will assist with the design of much needed tools for infection control, ultimately working towards malaria elimination.

## Contributors

TGC and SC conceived and directed the project. SS, AC, ATC, DF, GVT, FN, KS, DN, CJS, JD, MSM, DBP, CM, ARSB, RLDM, and SMS organised sample collection and processing. AI, MC, and SC undertook laboratory work including sequencing. AI and EM performed bioinformatic analysis under the supervision of SC and TGC, and together they interpreted the results. EDB provided software. AI, TGC, and SC wrote the first draft of the manuscript. All authors commented on the results and on the manuscript and approved the final submission.

## Data sharing statement

Raw sequence data is available from the European Nucleotide Archive under project code PRJEB56411 (see Table S2 for accession numbers of novel Brazilian isolates). The dataset also includes sequenced isolates from the MalariaGEN *P. vivax* Genome Variation project and described elsewhere.^32^^,^^34^^,^^40^^,^^42^, ^43^, ^44^

## Editor note

The Lancet Group takes a neutral position with respect to territorial claims in published maps and institutional affiliations.

## Declaration of interests

The authors have declared that no competing interests exist.
