# Supplementary material for: Population-based genomic study of Plasmodium vivax malaria in seven Brazilian states and across South America
Source: Lancet Reg Health Am. 2023 Jan 2;18:100420. doi: 10.1016/j.lana.2022.100420 (PMC9950661; doi:10.1016/j.lana.2022.100420)
Supplement: Supplementary Figs. S1–S11 [file mmc2.docx]

“Editor note: The Lancet Group takes a neutral position with respect to territorial claims in published maps and institutional affiliations.”

**SUPPLEMENTARY INFORMATION**

**SUPPLEMENTARY FIGURES**


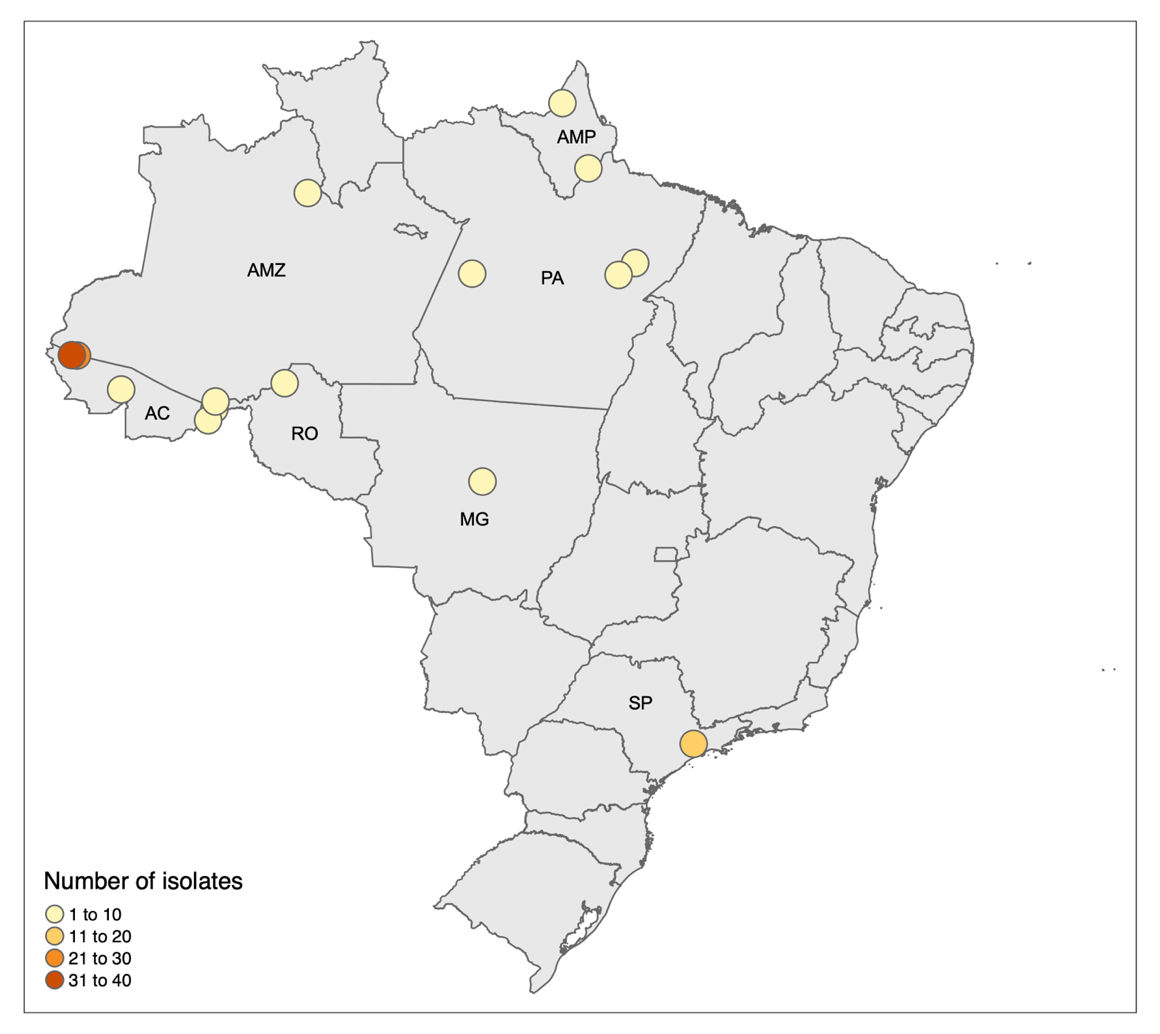


**S1 Figure. Location of Brazilian *Plasmodium vivax* isolates.**

The locations of the 123 Brazilian *P. vivax* isolates included within the final filtered database are plotted on a map of Brazil with the states where isolates were obtained labelled (AC; Acre, AMZ; Amazonas, RO; Rondônia, AMP; Amapá, PA; Pará, SP; São Paulo). Location points are coloured according to the scale, which represents the number of isolates from each location point. The location with the most isolates was Mancio Lima in Acre state (N = 38), and the lowest were Macapá within Amapá (N = 1) and Remansinho in Amazonas state (N = 1). For specific isolate location, date of collection and the corresponding accession numbers, please refer to **S2 Table**.


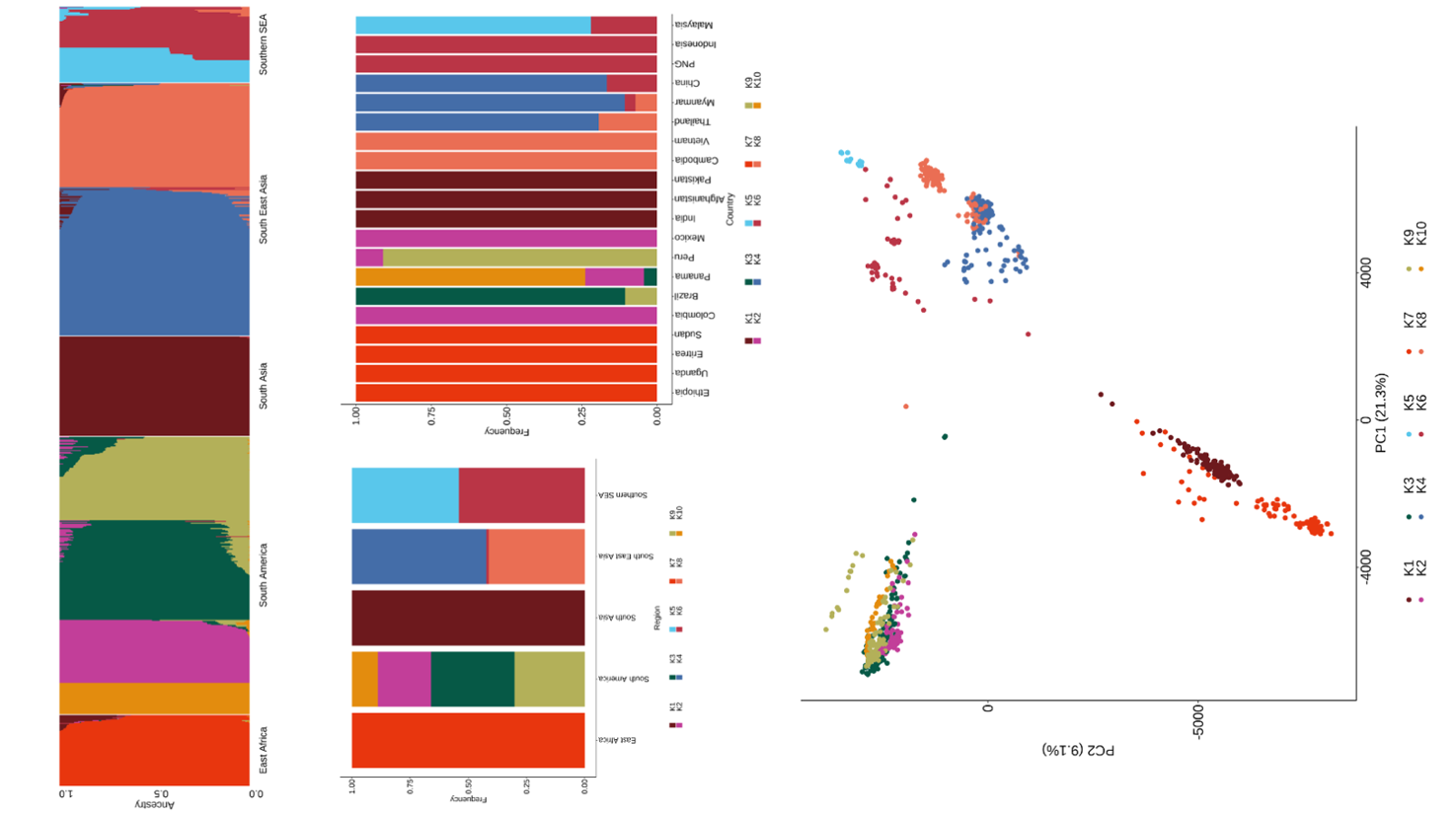


**S2 Figure. Admixture analysis**

All global isolates (n = 855) were assessed for population structure using admixture. Ten ancestral populations were assigned to the global dataset, the distribution of each ancestral population amongst each region **(A, B)** and country **(C)** is highlighted in the boxplots below. The PCA generated using the global SNP matrix is coloured by ancestral populations **(D).**

**
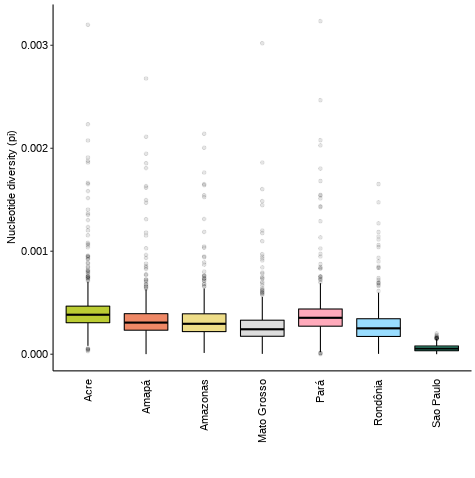
**

**S3 Figure. Nucleotide diversity within each state in Brazil**

Boxplot highlighting nucleotide diversity (π), calculated in sliding windows of 25 kbp across the genome using VCFtools for isolates within each state (Pará, n = 13; Amapá, n = 10; Mato Grosso, n = 5; Rondônia, n = 5; Acre, n = 74; Amazonas, n = 4; São Paulo, n = 12). All boxplot boxes consist of the median and interquartile range of the data for each country, with whiskers extending to extreme data points within 1.5 times the interquartile range from the box, data points beyond this range are outliers and plotted as points.

**
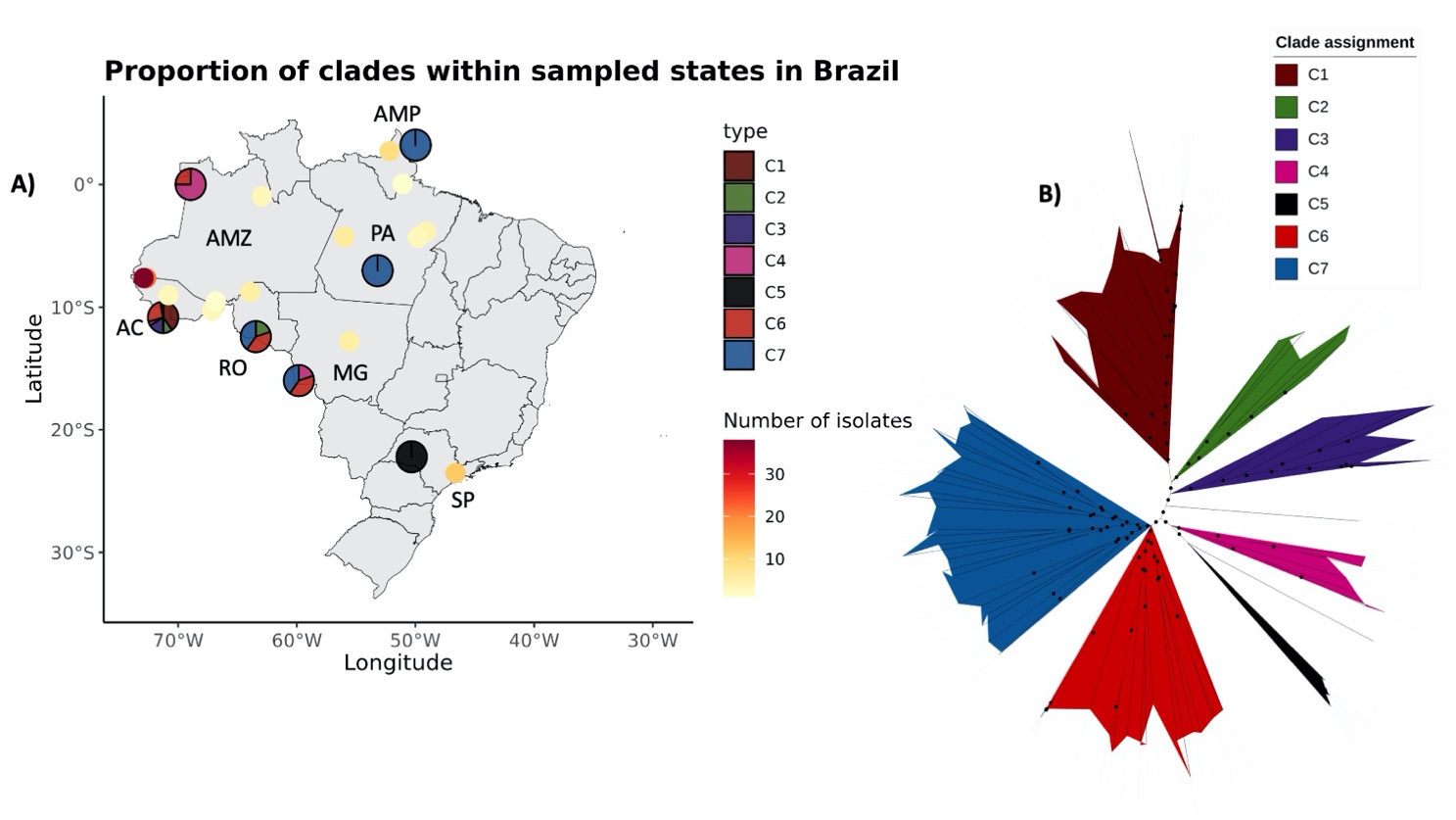
**

**S4 Figure. Proportion of assigned clades across sampled states in Brazil**

**A)** Map of Brazil with isolate location plotted as points, coloured according to the number of isolates sequenced at this location. States where isolates were obtained are labelled on the map (AC; Acre, AMZ; Amazonas, RO; Rondônia, MG; Mato Grosso, PA*;* Pará*,* AMP; Amapá, SP; São Paulo). Pie charts indicate the proportion of each clade found within each state. **B)** The Maximum Likelihood phylogenetic tree of 123 isolates from Brazil, comprising 70,757 SNPs, demonstrating the 7 clades assigned within the Brazilian dataset.


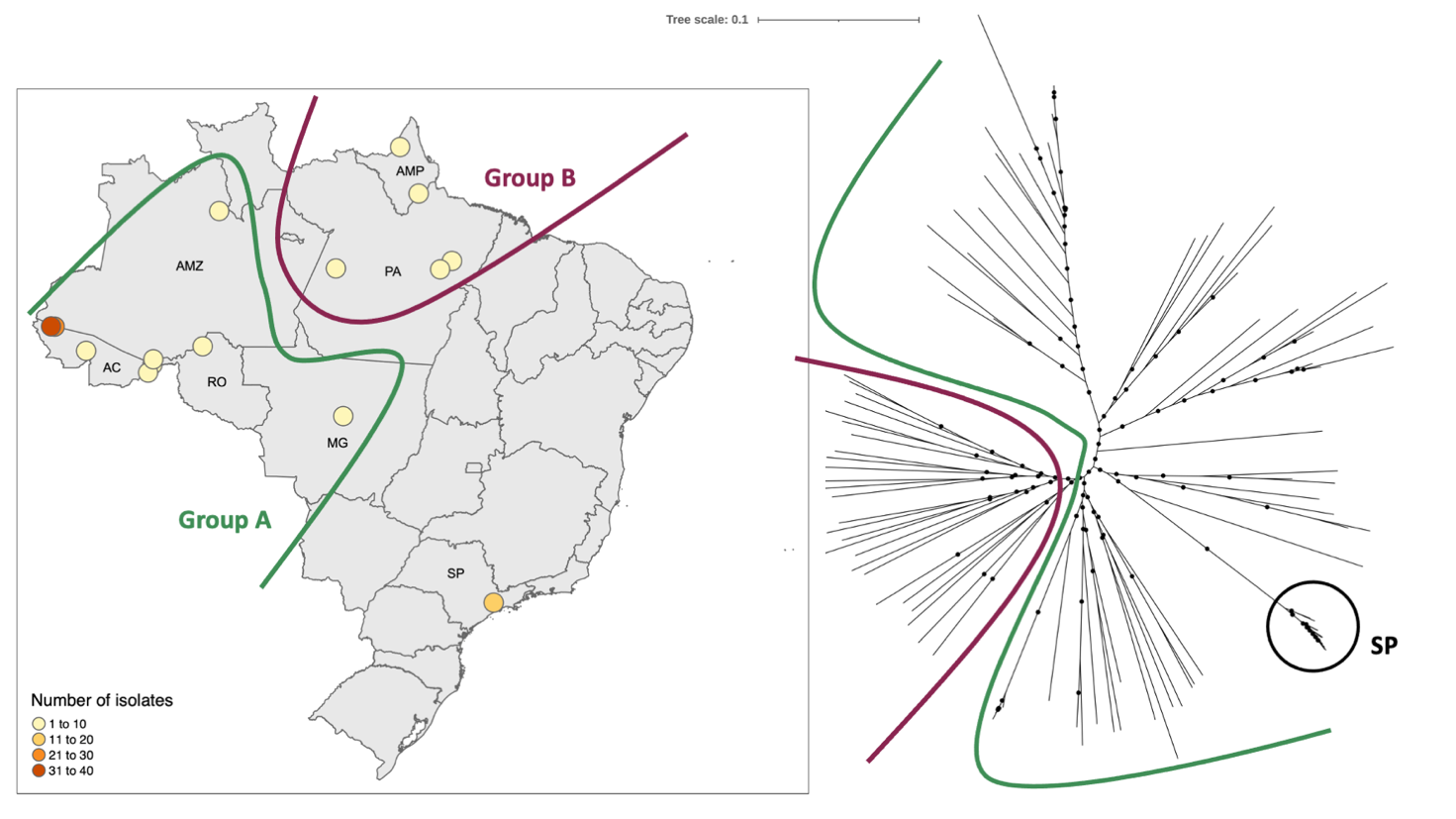


**S5 Figure. Brazilian isolates split into two population groups: Group A and Group B.**

A) The location of all Brazilian isolates within the filtered genomics database are shown on a map of Brazil with the states where isolates were obtained labelled (AC; Acre, AMZ; Amazonas, RO; Rondônia, MG; Mato Grosso, PA; Pará, AMP; Amapá, SP; São Paulo). A green border separates the Southwestern portion of the map where isolates are assigned into Group A. A purple border highlights the northern portion of Brazil where isolates are assigned to Group B. B) The Maximum Likelihood phylogenetic tree of 123 isolates from Brazil, comprising 70,757 SNPs with the corresponding borders to highlight isolates from group A (green border, N = 88 isolates) and group B (purple border, N = 23 isolates). Isolates from São Paulo are highlighted in a black circle labelled ‘SP’ and are excluded from these population groupings.


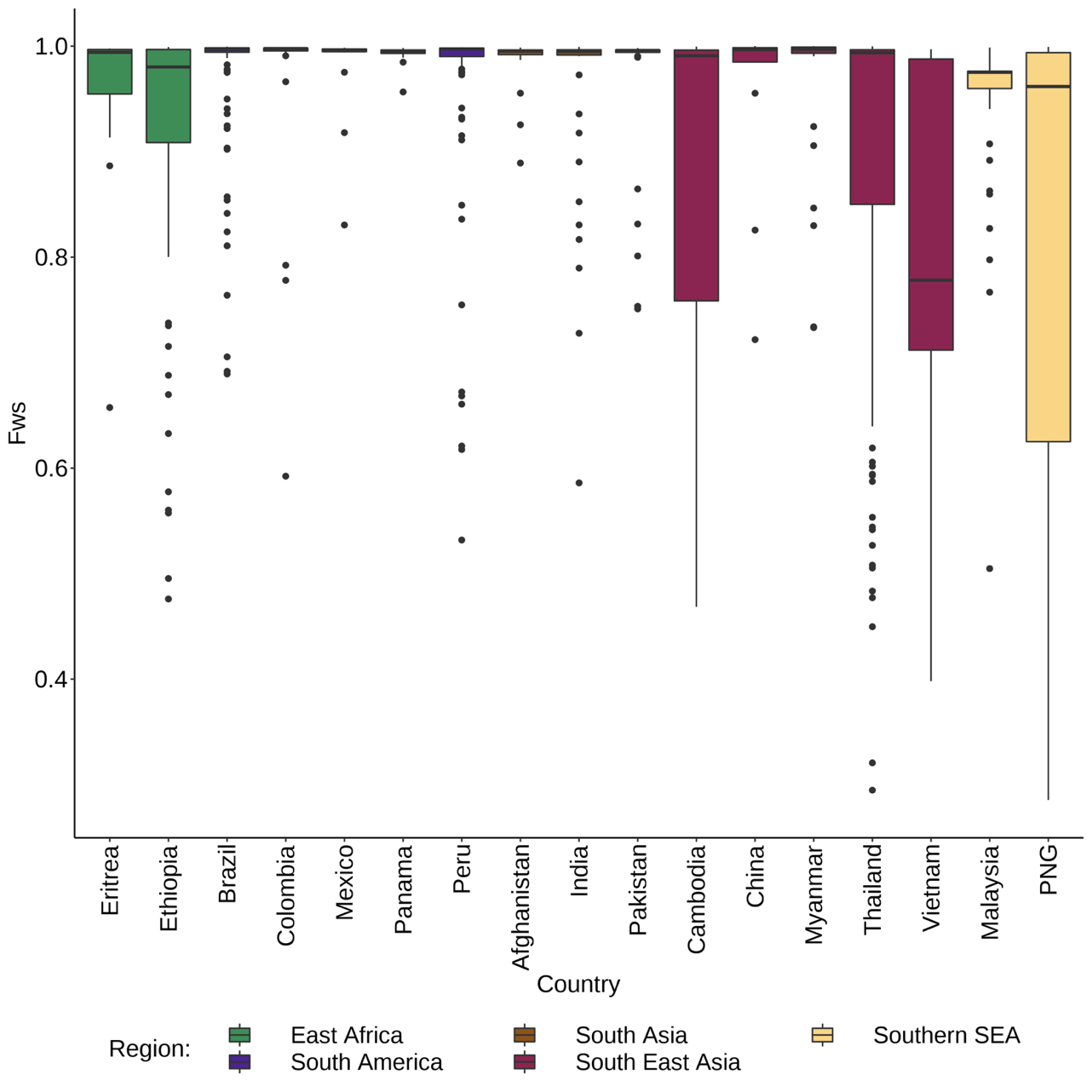


**S6 Figure. Multiplicity of infection within global isolates by country.**

Boxplot representing F_WS_ for *P. vivax* isolates across all countries with more than 10 isolates. Isolates are grouped by country and coloured according to region. All boxplot boxes consist of the median and interquartile range of the data for each country, with whiskers extending to extreme data points within 1.5 times the interquartile range from the box, data points beyond this range are outliers and plotted as points.

**
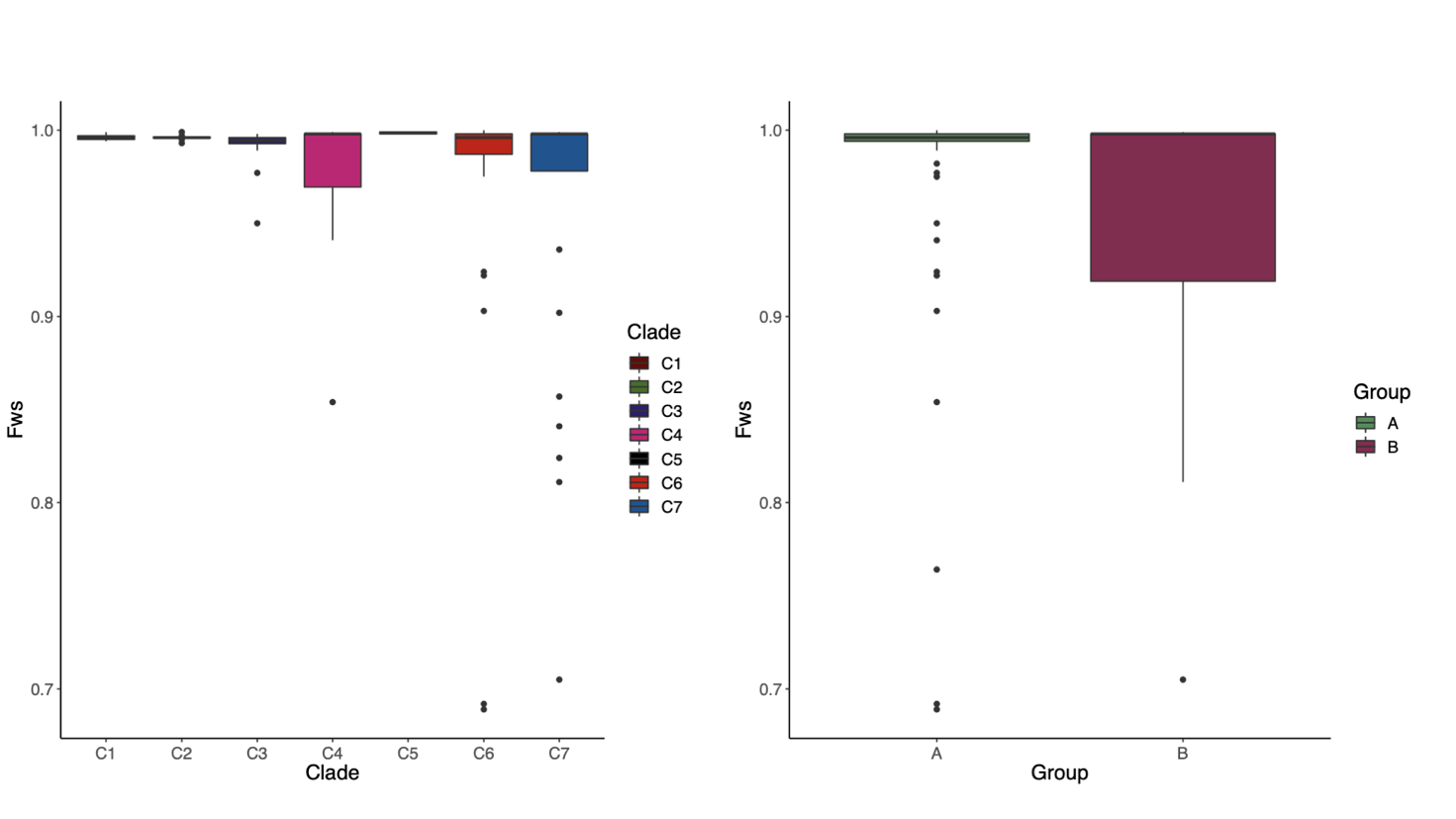
**

**S7 Figure Multiplicity of infection in isolates within Brazil groupings.**

Boxplot representing F_WS_ for Brazilian *P. vivax* isolates split into **A)** Clade groupings 1-7 and **B)** Geographical groupings A and B. All boxplot boxes consist of the median and interquartile range of the data for each country, with whiskers extending to extreme data points within 1.5 times the interquartile range from the box, data points beyond this range are outliers and plotted as points.


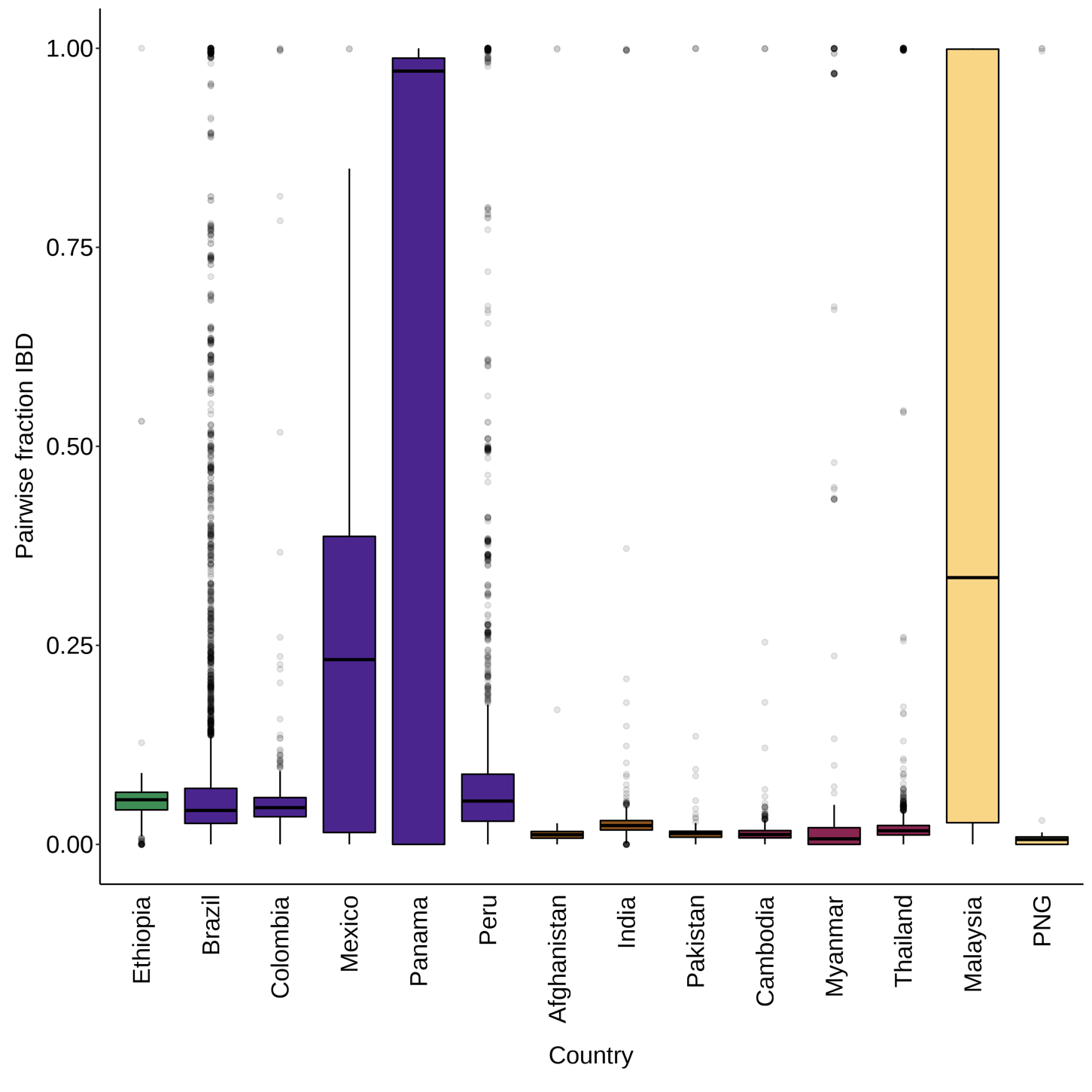


**S8 Figure. Pairwise IBD fractions within each country’s *P. vivax* population.**

Pairwise comparisons of IBD fractions amongst all isolates within each country (where there are >10 isolates per country). All boxplot boxes consist of the median and interquartile range of the data for each country, with whiskers extending to extreme data points within 1.5 times the interquartile range from the box, data points beyond this range are outliers and plotted as points. Median IBD value for each country, and the total number of isolates for each country included in IBD analysis is summarised in **S9 Table**. Boxplot boxes are coloured according to the assigned geographical region (East Africa = green, South America = purple, South Asia = brown, Southeast Asia = pink, Southern Southeast Asia = yellow).


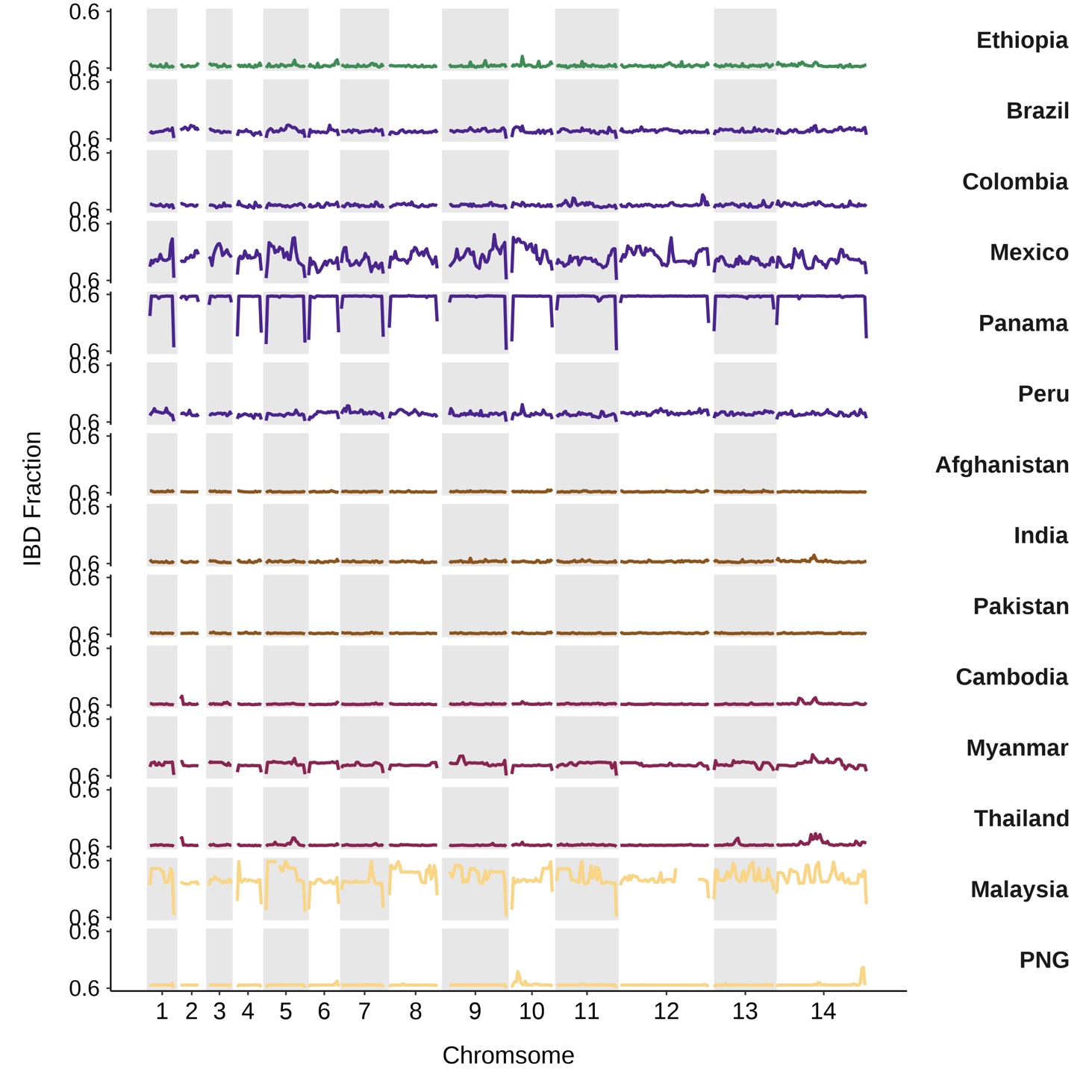


**S9 Figure. Genome-wide analysis of IBD fractions across each country.**

Using a sliding window of 50 kb, the genome wide IBD fractions are summarised for each country with >10 isolates. Line graphs are coloured according to the assigned geographical region (East Africa = green, South America = purple, South Asia = brown, Southeast Asia = pink, Southern Southeast Asia = yellow). Genome regions containing the top 1% of IBD fractions for each country are highlighted in **S8 Table**.

**
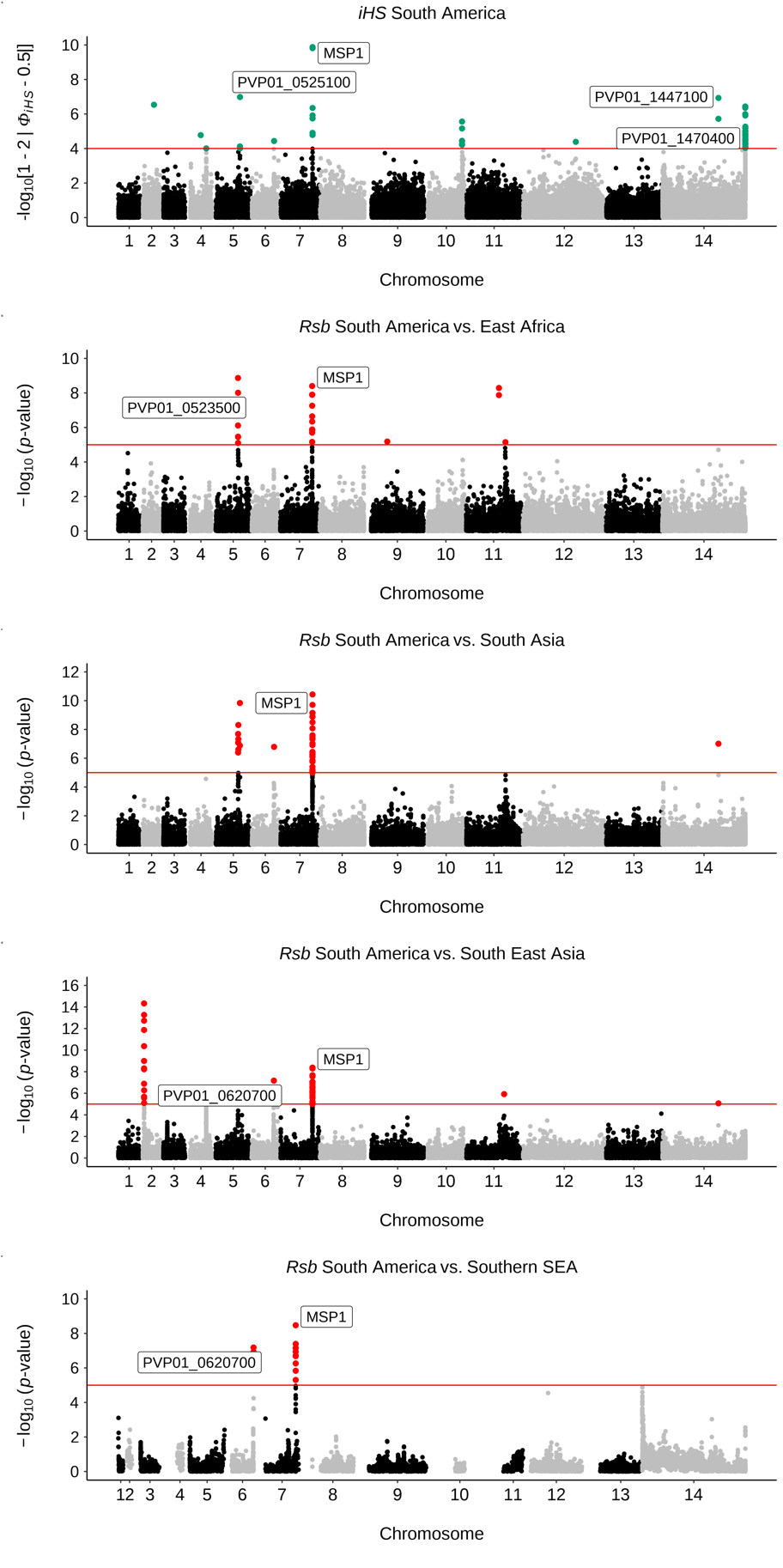
**

**S9 Figure. Selection signals (iHS) across the global dataset at the regional level.**

**
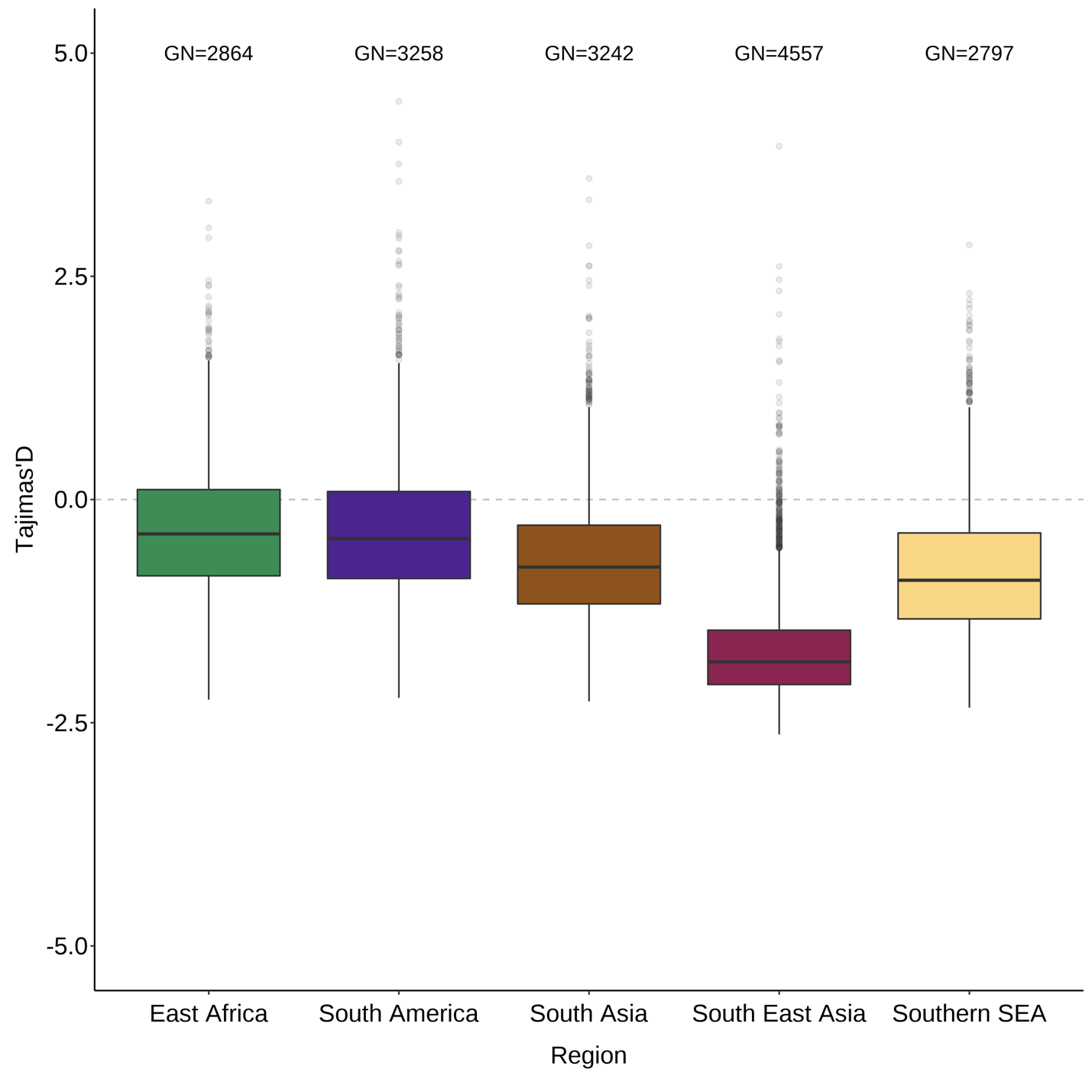
**

**S10 Figure. Distribution of Tajima’s D metric across all global regions.**

Boxplot demonstrating the distribution of Tajima’s D metric in each global region. Boxes are colour coded according to the geographical region (green = East Africa, purple = South America, brown = South Asia, pink = SEA, cream = SSEA) with the number of genes used in the Tajima D calculation (number of genes with > 5 SNPs in monoclonal isolates) is annotated at the top.

**
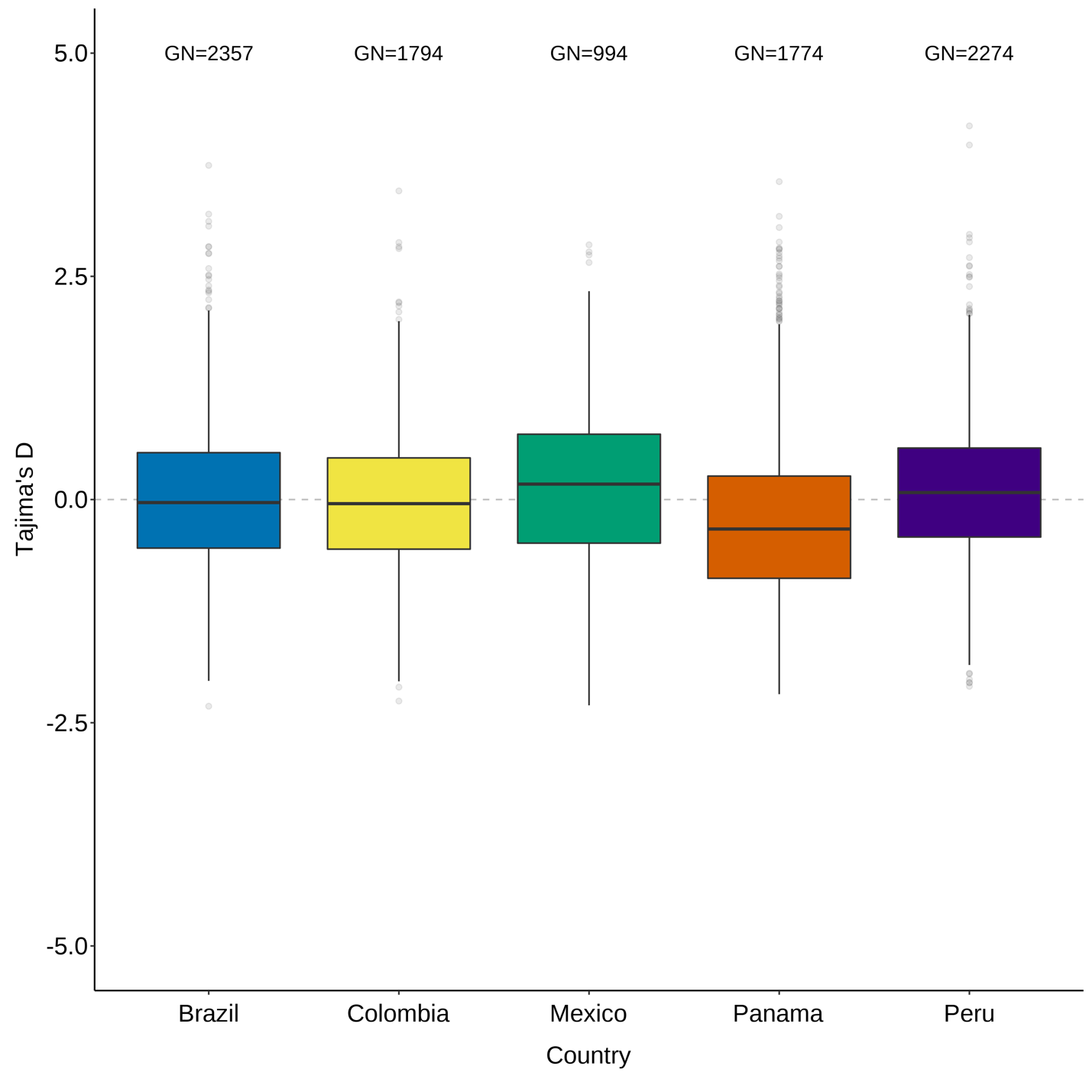
**

**S11 Figure. Distribution of Tajima’s D metric across all countries within South America.**

Boxplot demonstrating the distribution of Tajima’s D metric in each global region. Boxes are colour coded according to the country within South America (blue = Brazil, yellow = Colombia, green = Mexico, orange = Panama, purple = Peru) with the number of genes used in the Tajima D calculation (number of genes with > 5 SNPs in monoclonal isolates) is annotated at the top.
